# Investigation of recombination-intense viral groups and their genes in the Earth’s virome

**DOI:** 10.1038/s41598-018-29272-2

**Published:** 2018-07-31

**Authors:** Jan P. Meier-Kolthoff, Jumpei Uchiyama, Hiroko Yahara, David Paez-Espino, Koji Yahara

**Affiliations:** 1 Department of Bioinformatics, Leibniz Institute DSMZ – German Collection of Microorganisms and Cell Cultures, 38124 Braunschweig, Germany; 2 School of Veterinary Medicine, Azabu University, Sagamihara, Kanagawa 252-0206 Japan; 3 Department of Cell Signaling, Graduate School of Medical and Dental Sciences, Tokyo Medical and Dental University, Yushima 1-5-45, Bunkyo-ku, Tokyo 113-8549 Japan; 4 Department of Energy, Joint Genome Institute, Walnut Creek, California 94598 USA; 5 Antimicrobial Resistance Research Center, National Institute of Infectious Diseases, Higashimurayama, Tokyo 208-0011 Japan

## Abstract

Bacteriophages (phages), or bacterial viruses, are the most abundant and diverse biological entities that impact the global ecosystem. Recent advances in metagenomics have revealed their rampant abundance in the biosphere. A fundamental aspect of bacteriophages that remains unexplored in metagenomic data is the process of recombination as a driving force in evolution that occurs among different viruses within the same bacterial host. Here, we systematically examined signatures of recombination in every gene from 211 species-level viral groups in a recently obtained dataset of the Earth’s virome that contain corresponding information on the host bacterial species. Our study revealed that signatures of recombination are widespread (84%) among the diverse viral groups. We identified 25 recombination-intense viral groups, widely distributed across the viral taxonomy, and present in bacterial species living in the human oral cavity. We also revealed a significant inverse association between the recombination-intense viral groups and Type II restriction endonucleases, that could be effective in reducing recombination among phages in a cell. Furthermore, we identified recombination-intense genes that are significantly enriched for encoding phage morphogenesis proteins. Changes in the viral genomic sequence by recombination may be important to escape cleavage by the host bacterial immune systems.

## Introduction

Bacteriophages (phages), or bacterial viruses, are the most abundant and diverse group of biological entities on the planet^1,2^. Their abundance and lytic lifecycle impact global ecosystems, including nutrient and energy cycles as well as structures of microbial communities^3–5^. Temperate phages, which not only can integrate their genomes into their host’s chromosome but also transfer bacterial DNA to a new host by transduction, alter the biology of their hosts by introducing novel functions, such as virulence factors and drug resistance^6^. As a result, phages impact bacterial genome evolution and ecosystem biogeochemistry. Recent metagenomic studies have shown that phages are dominant members in the human microbiome and are suggested to have potential roles in maintaining health^7,8^. Thus, phages are of great interest in a number of disciplines such as medicine, virology, ecology, medicine, and environmental sciences.

Metagenomics have also revealed that a large virome is present anywhere in the biosphere, which is primarily composed of phages (i.e., the phageome)^9^. Moreover, the current classification of bacterial and archaeal viruses, curated by the International Committee on Taxonomy of Viruses (ICTV), only reflects a fraction of viral diversity; in particular, those currently present in public databases^10^. A recent, large-scale study of the Earth’s virome analyzed a huge amount of metagenomic sequence data from 3,042 geographically diverse samples to assess the global distribution, phylogenetic diversity, and host specificity of viruses^11^. The study discovered over 125,000 partial DNA viral genomes, increased the number of viral genes registered in public databases by 16-fold, and linked species-level viral groups to bacterial hosts using CRISPR spacers and transfer RNA matches^12,13^. The data have been utilized to develop a bacteria-phage interaction database^14^ and a bioinformatics pipeline for metagenomics-virus sequence discovery and virus clustering^15^.

Recombination is a fundamental driving force in evolution^16–19^, and occurs among different viruses inside the same bacterial host (e.g., co-infecting invasive viruses^20^, temperate phages and defective prophages^21^, or an invasive virus and a resident prophage^21^). Although phage genomes are generally known to be mosaic, with active recombination or horizontal genetic exchange^22^, recombination does not necessarily increase the average fitness of offspring^23,24^. It remains unexplored, in the rapidly increasing metagenomic data, whether such signatures of recombination are observed across various phylogenetic groups of phages. In addition, it is also unclear whether specific phylogenetic groups, or genes, are recombination-intense and show signatures of increased recombination due to natural selection. Thus, the fundamental aspect of recombination among phage genomes based on metagenomic data should be explored.

Here, we systematically examined signatures of recombination in every gene from 211 species-level viral groups in the Earth’s virome dataset that contain corresponding information on host bacterial species; (i) we determined recombination-intense viral groups throughout the virome dataset, (ii) we examined the relationship between recombination-intense viral groups and the potential strength of host immunity, and (iii) we closely examined the recombination-intense genes of the viral groups.

## Results

### Signatures of recombination are widespread among diverse viral groups

**Figure 1:**
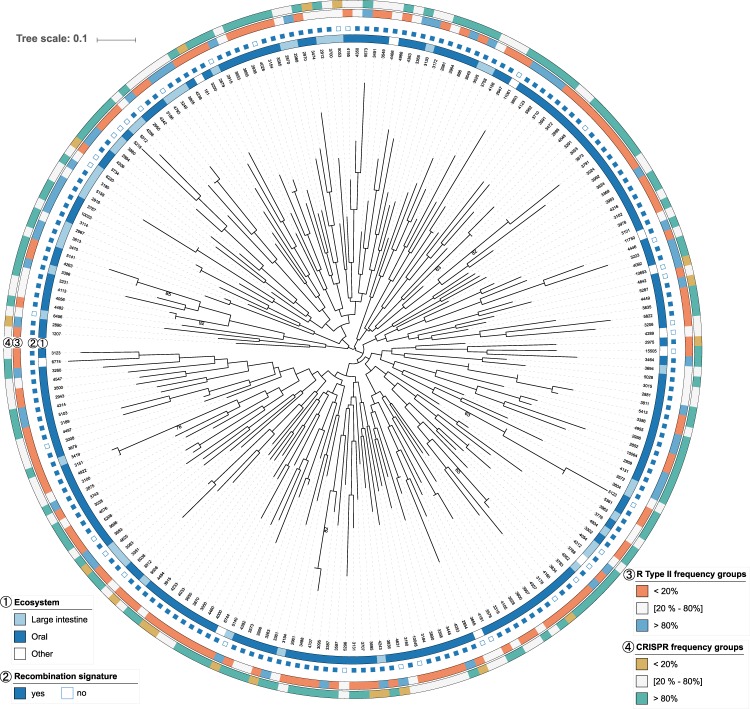
Proteome-based VICTOR tree of the 211 viral groups. Scale bar indicates interproteomic distance inferred using the distance formula *d*_4_. Viral group IDs defined in the previous study^11^ are shown as leaf labels. The innermost colored ring (1) indicates oral and intestinal samples, respectively. The next ring (2) indicates the presence or absence of recombination signatures (i.e., minimum number of recombination events ≥1). The two outer rings (3,4) indicate frequency categories for Type II restriction endonucleases (3) and CRISPR arrays (4) in a host bacterial species.

Among the more than 19,000 species-level viral groups defined in the Earth’s virome dataset composed of 3,042 metagenomic samples, we identified 211 viral groups that are usable for examining signatures of recombination and contain information on host bacterial species (Table S1). A proteomic tree of the 211 viral groups, constructed using the Virus Classification and Tree Building Online Resource (VICTOR) method^10^, is shown in Fig. 1, and a 16S rRNA gene maximum likelihood (ML) tree of the viral groups’ host bacterial species is shown in Fig. S1. The VICTOR tree (Fig. 1) revealed the presence of a very diverse dataset with long branches, almost no branch support (except for a few groups close to the tips of the tree), and an overall low phylogenetic signal. The host bacterial ML tree, on average, was well supported and reflected several major lineages (i.e., strains belonging to the phyla *Firmicutes*, *Fusobacteria*, *Proteobacteria*, and *Bacteroidetes*). About 75% of the viral groups originate from oral samples (Fig. 1) and account for 13.5% of all the metagenomic samples. For each viral group, we conducted a pan-genomic analysis and calculated the minimum number of recombination events (*r*_*min*_) for every orthologous gene using the four-gamete test, a conservative method to locate pairs of closest segregating sites within 4 haplotypes that are likely to be generated by recombination between them. As a result, 88% of the viral groups originating from oral samples showed at least one recombination event per gene (Fig. 1), whereas the proportion was 69% of viral groups originating from other samples.

### Recombination-intense viral groups

**Figure 2:**
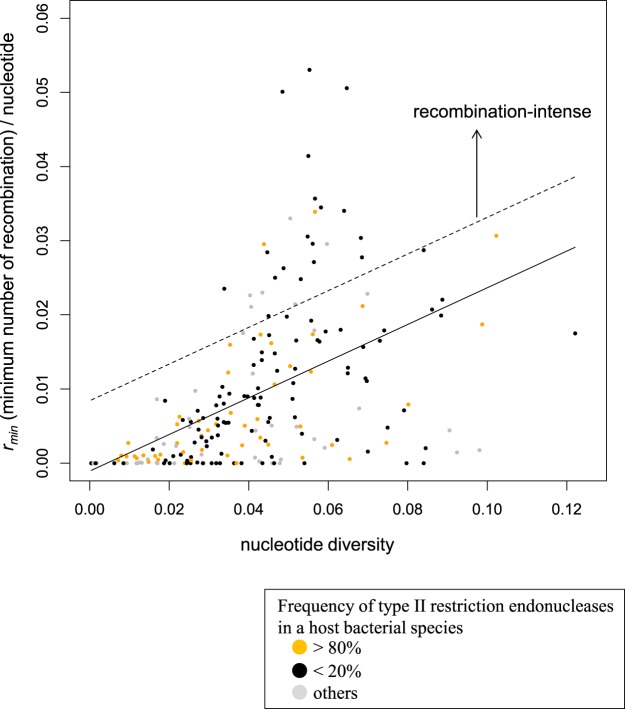
Relationship between the minimum number of recombination events (*r*_*min*_) per gene length and nucleotide diversity among the 211 viral groups. The x-axis and y-axis are nucleotide diversity and *r*_*min*_ per nucleotide, respectively. The solid line indicates the linear regression controlling for the number of sequences in each viral group. The dashed line indicates the threshold to focus on recombination-intense viral groups that considerably deviate from the regression line.

Next, we conducted a more quantitative analysis to explore the rates of recombination among viral groups. In order to account for the dependency of the *r*_*min*_ on gene length and nucleotide diversity, we took an approach similar to a previous study^25^. We plotted *r*_*min*_ per nucleotide versus nucleotide diversity of a viral group (Fig. 2), and calculated a linear regression line that captures the overall relationship between nucleotide diversity and the minimum number of recombination events per nucleotide. Using this relationship, we identified viral groups as recombination-intense that substantially deviate from the regression line. Based on the distribution of the deviation from the regression line among the 211 groups (Fig. S2), we identified the top 25 recombination-intense viral groups (above the dashed lines in Fig. 2 and Fig. S2, respectively). The host bacterial species of the 25 recombination-intense viral groups are shown in Table 1.

**Table 1:** Recombination-intense viral groups.

ID	Sample type	Host bacterial species	Nucleotide diversity	*r*_*min*_ (minimum number of recombinations)/nucleotide
3050	Oral (tongue dorsum)	*Atopobium* sp. ICM42b	0.055	0.053
181	Nasopharynx	*Propionibacterium acnes*	0.048	0.050
2961	Oral (tongue dorsum)	*Streptococcus mitis*	0.065	0.051
4000	Oral (tongue dorsum)	*Atopobium* sp. ICM42b	0.055	0.041
3707	Oral (tongue dorsum)	*Atopobium* sp. ICM42b	0.057	0.036
3591	Oral (supragingival plaque)	*Actinomyces gerencseriae*	0.058	0.035
3319	Oral (buccal mucosa)	*Gemella haemolysans*	0.050	0.033
3549	Oral (tongue dorsum)	*Prevotella nanceiensis*	0.064	0.034
3189	Oral (supragingival plaque)	*Leptotrichia goodfellowii*	0.056	0.030
2890	Oral (supragingival plaque)	*Capnocytophaga granulosa*	0.057	0.034
2943	Oral (tongue dorsum)	*Campylobacter concisus*	0.068	0.030
3600	Oral (attached/keratinized gingiva)	*Gemella haemolysans*	0.060	0.030
3200	Oral (buccal mucosa)	*Streptococcus oligofermentans*	0.049	0.026
3358	Oral (tongue dorsum)	*Streptococcus gordonii*	0.047	0.025
4460	Oral (tongue dorsum)	*Atopobium* sp. ICM42b	0.056	0.027
3915	Oral (buccal mucosa)	*Gemella haemolysans*	0.040	0.023
3836	Oral (tongue dorsum)	*Oribacterium sinus*	0.069	0.028
3445	Oral (buccal mucosa)	*Streptococcus mitis*	0.053	0.025
2970	Oral (supragingival plaque)	*Capnocytophaga* sp. CM59	0.055	0.031
2959	Oral (buccal mucosa)	*Actinomyces viscosus*	0.044	0.030
3184	Oral (tongue dorsum)	*Streptococcus mitis*	0.045	0.028
3776	Oral (tongue dorsum)	*Prevotella denticola*	0.052	0.021
2981	Oral (tongue dorsum)	*Lachnoanaerobaculum saburreum*	0.041	0.021
3576	Oral (tongue dorsum)	*Mogibacterium* sp. CM50	0.045	0.020
4484	Oral (tongue dorsum)	*Lachnospiraceae* bacterium oral taxon 082	0.084	0.029

Ordered by the extent of deviation from regression.

Almost all (24 out of 25) the recombination-intense viral groups were from oral samples, which is a statistically significant enrichment (*P* = 0.0066, Fisher’s exact test). Only viral group 181 originated from nasopharynx samples, but its host bacterial species, *Propionibacterium acnes*, forms part of the normal flora in the human oral cavity^26^. Namely, all the recombination-intense viral groups infect bacterial species that reside in the human oral cavity.

For the recombination-intense viral groups, we mapped reads back to the genomes to check their coverage (Fig. S3). Overall, the reads were mapped to the entire genome sequences, and the mean coverage per viral group was 24, eliminating possibilities that the elevated rates of recombination among the viral groups were primarily due to low coverage and metagenome mis-assembly in these sequences. There were a few viral groups showing some unusual patterns: sporadic low coverage typically in intergenic regions (viral groups 3050, 3776, 3576, 181, 3549, and 4000), and coverage difference along the genome (e.g. higher coverage at the 5′ end of viral group 4460 or 3184, and at the 3′ end of viral group 3200). The intergenic regions and the 3′ end of viral group 3200 clearly do not influence the results because they were not included in our pan-genomic analysis of orthologous genes. Regarding the higher coverage at the 5′ end of viral group 4460 or 3184, we confirmed that rates of recombination at the gene level (explained below in “Notable recombination-intense genes”) did not show significant increase in the regions (*P* = 1 and *P* = 0.33, one-sided Welch’s t-test).

### Relationship between recombination-intense viral groups and host immunity

In order to deepen our understanding of species-level relationships between viral groups and their hosts, we examined the frequency of each host bacterial species’ immune systems (i.e., restriction-modification and CRISPR-Cas), self-protective mechanisms that cleave phage DNA when injected into a bacterial cell^27^. Type II restriction endonucleases, which recognize a specific nucleotide sequence and cleave at a fixed position^28^, and CRISPR arrays, which store the immunological memory of invading pathogens^29^, were included in the study. We classified three frequency categories (**1:** <20%, **2:** between 20% and 80%, **3:** >80%) of host immunity, which are shown in the outer rings of Fig. 1. As a result, we found a statistically significant inverse association between the recombination-intense viral groups and Type II restriction endonucleases; host bacteria species having Type II endonucleases at a frequency >80% (orange dots in Fig. 2) are rarely found (2/25) among the recombination-intense groups, compared to the other viral groups (*P* < 0.05 Fisher’s exact test). The result was the same when we used cutoff frequency 70% or 60%.There were only two such host bacterial species in the recombination-intense viral groups (*Capnocytophaga granulosa* and *Actinomyces viscosus*). This suggests that the high frequency of Type II endonucleases in host bacterial species could be effective in cleaving invading phages and reducing opportunities for recombination among phages in a cell. Furthermore, we did not find any other significant associations for either Type I or III restriction endonucleases, or CRISPR arrays in host bacterial species.

**Figure 3:**
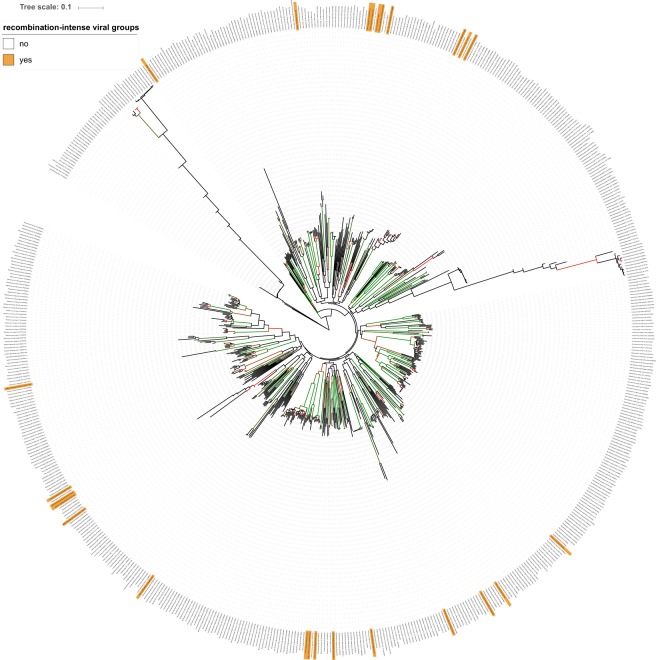
Phage proteomic tree based on the VICTOR method using a united dataset of comprehensive ICTV reference data and the recombination-intense viral groups. Leaf labels representing the recombination-intense viral groups are highlighted in orange. Branch support is indicated by color from red (50%) to green (100%). The vicinity of these metagenomic samples to actual ICTV phage species provides hints regarding their composition. Scale bar indicates interproteomic distances calculated via the distance formula *d*_4_. The tree was rooted at the midpoint^69^.

Additionally, we tried to reveal the phylogenetic nature of our metagenomic samples by conducting a joint phylogenetic analysis with an ICTV reference dataset using the VICTOR method^10^. The results of the analysis are shown in Fig. 3. Clearly, the recombination-intense viral groups are widely distributed across the ICTV taxonomy, even viral groups sharing the same (assumed) host bacterial species are not closely located in the phylogenic tree, indicating a diverse composition of the underlying metagenomic samples. However, at least some of the recombination-intense viral groups from this study were found in a well-supported subtree (e.g. groups 3319, 3707 and 4460), indicating a rather high phylogenetic relation to nearby ICTV reference phages. As observed earlier, the backbone of the tree is only weakly supported which is due to the nature of such diverse phage genomic datasets and is likely caused by multiple origins of prokaryotic viruses^10,30^.

### Notable recombination-intense genes

**Figure 4:**
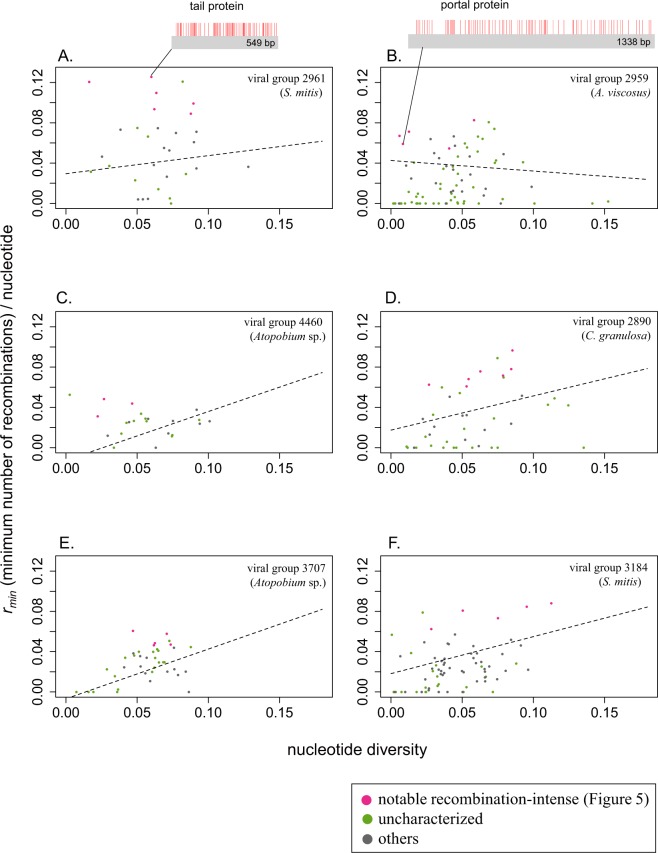
Examples of notable recombination-intense genes deviating from the regression of *r*_*min*_ per gene length on nucleotide diversity in each viral group. The x-axis and y-axis are the same as in Fig. 2. Pink: notable recombination-intense genes (Fig. 5 and Table S2). Green: uncharacterized genes. Gray: others. Recombination breakpoints in a phage tail gene and a portal gene are shown as red vertical bars at the top. The dashed line indicates the linear regression controlling for the number of sequences in each gene.

We further explored recombination-intense genes by similarly analyzing the relationship between *r*_*min*_ per nucleotide and nucleotide diversity at the gene level in each recombination-intense viral group (Figs 4 and S5). Substantial deviations from the regression were found; for example, in a gene encoding a phage tail protein (Fig. 4A) and a gene encoding a phage portal protein (Fig. 4B), with recombination breakpoints found throughout both genes. Because approximately 90% of such genes were initially annotated as hypothetical, we conducted iterative protein searches based on representing both query and database sequences by profile hidden Markov models^31^ using a UniProt database. We examined all hits with >99% probability of being true positives, and identified 89 notable genes in 24 viral groups (listed in Table S2).

**Figure 5:**
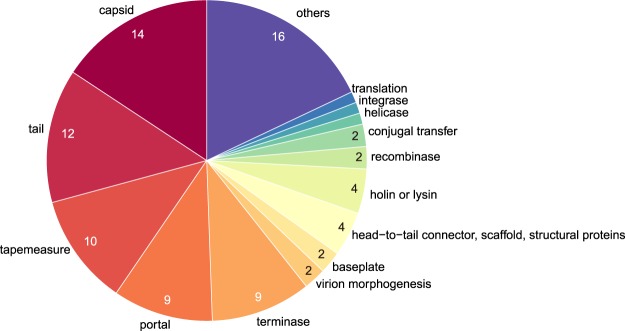
Breakdown of notable recombination-intense genes. The number of genes in each category is shown in the pie chart. More detailed information on each gene is shown in Table S2.

A breakdown of the notable recombination-intense genes is shown in Fig. 5. Approximately 75% of this set are genes that encode phage morphogenesis proteins. Among these, approximately 19% are associated with head morphogenesis, including genes for capsid, virion morphogenesis, and scaffold proteins^32^. Approximately 21% are associated with phage neck and DNA packaging, including portal protein, head-to-tail connector, and terminase^33,34^. Approximately 27% are associated with phage tail, including the genes for tail protein, tape measure protein, and baseplate^33,35,36^. Moreover, approximately 4% are DNA-associated genes, including the genes for integrase, helicase, and recombinase. Approximately 4% are lysis-associated genes, including holin and endolysin. Holin and endolysin are essential for host cell lysis in the lytic lifecycle^37^. Finally, the frequency of such genes in the pan-genome of other viral groups is approximately 27%, indicating significant enrichment among the notable recombination-intense genes (*P* < 10^−15^, chi-squared test).

## Discussion

The recombination events we detected and examined are those that have survived in the viral groups, and which are inevitably affected by natural selection^17^. These genes are likely to be under selective pressure from host bacterial immunity attacking foreign DNAs (i.e., restriction-modification and CRISPR-Cas systems), as this cleavage would effectively prevent survival and propagation of phages. These genes were found in the recombination-intense viral groups, all of which infect bacterial species living in human oral cavities, where hundreds of thousands of CRISPR spacer groups are transcribed^38^. Hence it is likely that there is a genetic conflict invoked by the coevolution of phages and host oral bacteria by means of classic Red Queen dynamics^39^. The signature of highly elevated recombination would reflect the evolutionary conflict in which phages continuously change their genomic sequences by recombination to escape cleavage by the host bacterial immune system in the human oral cavity. A recent study showed that acquisition of random mutations is not sufficient for phages to completely escape CRISPR-Cas targeting in a continuous co-culture^27^. Another study of long-term bacterium-phage coevolution experiments showed the presence of multiple phages increased phage persistence by enabling recombination-based formation of chimeric phage genomes in which sequences heavily targeted by CRISPR were replaced^40^. Therefore, recombination would play an important role, at least for recombination-intense oral phages, in escaping bacterial immunity.

Similarly, our analysis of Type II restriction endonucleases suggest that its high frequency in host bacterial species could be effective in cleaving invading phages and reducing opportunities for recombination among phages in a cell. This raises the interesting possibility that restriction-modification could be more effective than CRISPR-Cas in oral bacteria for dealing with phages. Recently, it was suggested that host bacteria are generally incapable of utilizing CRISPR-Cas to eradicate phages from the human oral cavity as well as the gut^38^. Further studies are warranted to elucidate the relative roles of restriction-modification and CRISPR-Cas in contributing to bacterial immunity. A recent study, in a different context, showed that CRISPR-Cas and restriction-modification function additively, at least against conjugative antibiotic resistance plasmid transfer in *Enterococcus faecalis*^41^.

Alternatively, the enrichment of recombination-intense viral groups in human oral cavities could be explained in terms of frequency of recombinases among the viral groups. However, there was no statistically significant difference in frequency of viral groups carrying a recombinase (*P* = 0.6, chi-square test) between the recombination-intense viral groups (38%) and the others (30%).

Regarding the bacterial host species of the recombination-intense viral groups (Table 1), many are recognized as part of the normal human oral flora, but sometimes cause disease. For example, some *Atopobium* species have been identified as agents of chronic periodontitis and bacteremia^42,43^. The viridans group streptococci (VGS), which consist of *Streptococcus mitis*, *Streptococcus gordonii*, and *Streptococcus oligofermentans*, can cause a wide range of infections in humans, including bacteremia, infective endocarditis, and moderate or severe clinical disease (e.g., VGS shock syndrome)^44^. Among others, associations with endocarditis have been reported for *Actinomyces viscosus*^45^, *Gemella haemolysans*^46^, and *Leptotrichia goodfellowii*^47^, while associations with bacteremia were reported for *Leptotrichia goodfellowii*^47^ and *Capnocytophaga* species^48^. In addition, *Campylobacter concisus* has been linked to prolonged diarrhea and inflammatory bowel disease^49^. Meanwhile, the phage lytic lifecycle has been shown to play a role in preventing outgrowth and dysbiosis by killing the bacterial host^39^. According to a recent review^50^, endogenous phages can play an important role in human oral health by limiting overgrowth of bacteria and maintaining the commensal microbiota at acceptable levels that can then be controlled by the human immune system. The evolution of recombination-intense phages could contribute to such maintenance function against oral host bacterial species during their coevolution.

Given these observations, the recombination-intense viral groups which are particularly present in oral viromes, could boost phage evolution and contribute to the maintenance of the commensal microbiota (eubiosis)^39^. This is the first systematic, quantitative study of recombination in phage genomes across >200 diverse viral groups, and the first study to explore viral-host relationships from a viewpoint of recombination and host immune systems.

## Materials and Methods

### Selection of viral groups and individual scaffolds

From 17,803 viral groups defined in the previous study^11^ as having ≥90% bidirectional average amino acid identity and ≥50% total alignment fraction, we extracted 211 groups consisting of at least four individuals (scaffolds) required for the examination of minimum number of recombination events and carrying information of host bacteria at the species level. The information was inferred in the previous study^11^ and is stored in IMG/VR database^13^ by either perfect matches of viral tRNAs, or matches of CRISPR-Cas spacers requiring at least 95% identity over the whole spacer length, and allowing only 1–2 SNPs at the 5′ end of the sequence.

Scaffold IDs for each individual in each viral group is listed in Table S3. We downloaded nucleotide sequences of the scaffolds through the ‘Expert Review’ version of IMG/M ER (https://img.jgi.doe.gov/mer/)^10^ datamart^12^. We made the nucleotide sequence data directly downloadable at https://figshare.com/articles/211viralgroups_fas_tgz/6223769.

### Proteomic tree of the viral groups

A proteomic tree of the 211 viral groups was constructed using the Virus Classification and Tree Building Online Resource (VICTOR) method, publicly available at https://victor.dsmz.de^10^. From the three distinct trees generated by VICTOR, the one based on distance formula *d*_4_ was chosen because this formula is robust^51^ when using incomplete genomes and represented the most reasonable choice in view of the partially incomplete nature of the metagenomic samples. The tree was finally visualized and annotated using the iTOL web service^52^.

### Pan-genome analysis of recombination and nucleotide diversity

The prediction of protein-coding genes and gene annotation was performed using Prokka^53^ software with the “-k Viruses” option. We conducted pan-genome analyses using the Roary^54^ pipeline with “-e --mafft -i 90 -z” options and obtained alignments of 12,319 orthologous genes. The minimum number of recombination events (*r*_*min*_) was calculated for each orthologous gene using the four-gamete test^55^ that locates pairs of closest segregating sites within 4 haplotypes that are likely to be generated by recombination between them. We used the method implemented in the PGEToolbox^56^, which filters gaps in advance. Basic population genetic statistics (e.g. nucleotide diversity) were also calculated for each orthologous gene using DnaSAM^57^.

### Annotation of host bacteria species

We downloaded nucleotide sequences of individual genomes and their annotations of the host bacterial species that are available in the MBGD database^58^ (http://mbgd.genome.ad.jp/htbin/getData?table=genome). For each host bacterial species, we checked the frequency of individuals that have either Type I, II, or III restriction enzymes, or CRISPR-arrays that were detected by CRISPRDetect^59^.

A 16S rRNA gene sequence phylogeny was inferred by the GGDC web server^51^, available at https://ggdc.dsmz.de/, using the DSMZ phylogenomics pipeline^60^ adapted to single genes. A multiple sequence alignment was created with MUSCLE^61^. Maximum likelihood (ML) and maximum parsimony (MP) trees were inferred from the alignment with RAxML^62^ and TNT^63^, respectively. For ML, rapid bootstrapping in conjunction with the autoMRE bootstrapping criterion^64^, and subsequent search for the best tree was used; for MP, 1000 bootstrapping replicates were used in conjunction with tree-bisection-and-reconnection branch swapping and 10 random sequence addition replicates. The sequences were checked for compositional bias using the Chi-squared test as implemented in PAUP*^65^.

### Identification and taxonomic assignments of recombination-intense viral groups

We calculated the sum of the *r*_*min*_ across the orthologous genes divided by the sum of their lengths, in base pairs, for each viral group. We also calculated nucleotide diversity of each orthologous gene using DnaSAM^57^, and its average across the orthologous genes for each viral group. We then conducted multiple linear regressions to capture the overall relationship between the *r*_*min*_ per nucleotide and nucleotide diversity after controlling for differences in the number of individuals in a viral group: ${y}_{i}={\beta }_{0}+{\beta }_{1}{x}_{1,{\rm{i}}}+{\beta }_{2}{x}_{2,{\rm{i}}}+{\epsilon }_{{\rm{i}}}$ where, for viral group *i*, *y*_*i*_ is the minimum number of recombination events per nucleotide; *x*_*1*,*i*_ is nucleotide diversity; *x*_*2*,*i*_ is the number of individuals; *β*_0_ is the intercept; *β*_1_ and *β*_2_ are regression coefficients; and ε_i_ is error, which is normally distributed. We plotted the regression line in Fig. 2 given the parameter estimates, holding constant *x*_2_ as the average number of individuals among the viral groups. Using this relationship, we identified the top 25 viral groups having >0.009 deviation from the regression line as recombination-intense. We chose the cutoff by examining the empirical distribution (Fig. S2) and looking for approximately top 10 percentile. A caveat is frequent recombination among very closely related sequences might not be identified by this approach because such recombined sequences are expected to be almost the same as their parental sequences, and in principle be difficult to be detected by comparison of nucleotide sequences.

The recombination-intense viral groups were added to a recently published ICTV reference dataset^10^ which together was used for the inference of a proteome-based tree via the VICTOR method^10^. In particular, pairwise distances were inferred from the distance formula *d*_4_ because this formula is robust when using incomplete sequences^10,51^.

### Identification and annotation of recombination-intense genes

For each recombination-intense viral group, we conducted multiple linear regressions, similar to above, but at the gene level rather than the viral group level: ${y}_{i}={\beta }_{0}+{\beta }_{1}{x}_{1,i}+{\beta }_{2}{x}_{2,i}+$ ε_i_ where, for gene *i* in a viral group, *y*_*i*_ is the minimum number of recombination events per nucleotide; *x*_*1*,*i*_ is nucleotide diversity; *x*_*2*,*i*_ is the number of aligned sequences; *β*_0_ is the intercept; *β*_1_ and *β*_2_ are regression coefficients; and ε_i_ is error, which is normally distributed. We plotted the regression lines in Fig. 4 and Fig. S5 given the parameter estimates, holding constant *x*_2_ as the average number of aligned sequences in a gene. For each gene, we translated the alignment and conducted iterative protein searches using HHblits^31^, which represents both query and database sequences by profile hidden Markov models (i.e., condensed representation of multiple sequence alignments specifying, for each sequence position, the probability of observing each of the 20 amino acids) instead of single sequences for the detection of remote homology. We used the clustered uniprot20_2016_02 database (http://wwwuser.gwdg.de/~compbiol/data/hhsuite/databases/hhsuite_dbs/), which covers essentially all of the sequence universe by clustering the UniProt database^66^ from EBI/SIB/PIR and the non-redundant (nr) database from the NCBI. For all hits with >99% probability of being true positives, we individually examined each annotation, and the extent of deviation from the regression line, to identify notable recombination-intense genes.

### Mapping reads back to genomes of recombination-intense viral groups

For each recombination-intense viral group carrying at least a notable recombination hot gene, we selected a representative with the longest possible scaffold, and mapping reads from their respective sample stored in back to it using Bowtie2^67^. Raw reads were collected from the Sequence Read Archive (SRA) according to their run and sample identifiers (shown in Table S1) obtained via Genomes OnLine Database (GOLD)^68^. Visualization and statistics (mean coverage and standard deviation) were obtained using Geneious software (Biomatters Ltd., Auckland, New Zealand). Visualization of the viral contigs after gene calling was obtained from the Integrated Microbial Genomes with Metagenomes (IMG/M) platform^12^. Genes are colored in the maps according with their predicted function (based on clusters of orthologous groups; COGs).

## Electronic supplementary material

Supplementary Information

Table S1

Table S2

Table S3
