# Supplementary material for: Investigation of recombination-intense viral groups and their genes in the Earth’s virome
Source: Sci Rep. 2018 Jul 31;8:11496. doi: 10.1038/s41598-018-29272-2 (PMC6068154; doi:10.1038/s41598-018-29272-2)
Supplement: Supplementary file 1 — Supplementary Information [file 41598_2018_29272_MOESM1_ESM.pdf]

Supplemental Information for:

Investigation of recombination-intense viral groups and their genes in the Earth's virome

Jan P. Meier-Kolthoff[1], Jumpei Uchiyama[2], Hiroko Yahara[3], David Paez-Espino[4], and Koji Yahara[5*]

[1] Department of Bioinformatics, Leibniz Institute DSMZ – German Collection of Microorganisms and Cell Cultures, 38124 Braunschweig, Germany

[2] School of Veterinary Medicine, Azabu University, Sagamihara, Kanagawa, 252-0206, Japan

[3] Department of Cell Signaling, Graduate School of Medical and Dental Sciences, Tokyo Medical and Dental University, Yushima 1-5-45, Bunkyo-ku, Tokyo, 113-8549, Japan

[4] Department of Energy, Joint Genome Institute, Walnut Creek, California 94598, USA

[5] Antimicrobial Resistance Research Center, National Institute of Infectious Diseases, Higashimurayama, Tokyo, 208-0011, Japan

[*]Corresponding author: Koji Yahara (k-yahara@nih.go.jp)

Supplementary Tables

Table S1, S2, and S3 are available as spreadsheets.

Supplementary figure legends

Figure S1. A 16S rRNA gene maximum likelihood tree of the viral groups' host bacterial species.

Figure S2. Distribution of the deviation from the regression line among the 211 viral groups. The dashed vertical line is the cutoff (> 0.009) to define the recombination-intense viral groups.

Figure S3. Mapping reads back to genomes of recombination-intense viral groups. For each viral group, a representative with the longest possible scaffold was used as a reference for the read mapping. Colors of genes are defined at the end according with their predicted function (based on clusters of orthologous groups; COGs).

Figure S4. Phage proteomic tree based on the VICTOR method using a united dataset of comprehensive ICTV reference data and the recombination-intense viral groups. The numbers in circles indicate the assignment of known phages to the ICTV classification at the species (1), genus (3), subfamily (5), and family (7) levels. The remaining numbers indicate the assignment to proteome-based species (2), genus (4), subfamily (6), and family clusters (8) as inferred by the VICTOR method. Leaf labels representing the recombination-intense viral groups are highlighted in orange. The vicinity of these metagenomic samples to actual ICTV phage species, genera, and (sub-)families provides hints regarding their composition. Scale bar indicates interproteomic distances calculated via the distance formula d_4. The tree was rooted at the midpoint[69].

Figure S5. Notable recombination-intense genes deviating from the regression of r_{min} per gene length on nucleotide diversity in each viral group. Viral groups not in Figure 4 are shown. The x-axis and y-axis are the same as in Figure 2 and Figure 4. Pink: notable recombination-intense genes (Figure 5 and Table S2). Green: uncharacterized genes. Gray: others. Recombination breakpoints in a phage tail gene and a portal gene are shown as red vertical bars at the top. The dashed line indicates the linear regression controlling for the number of sequences in each gene.

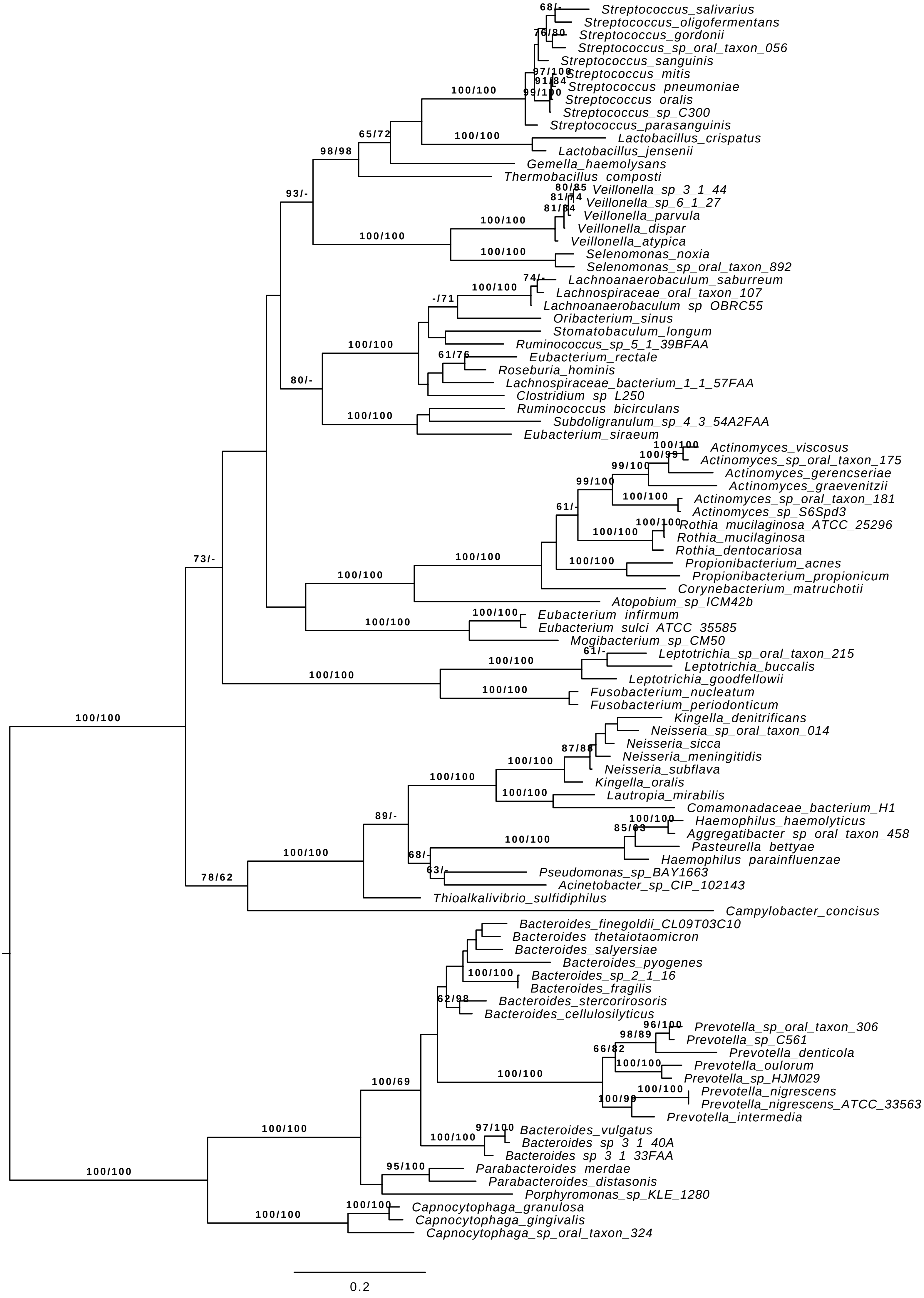
Streptococcus_salivarius
Streptococcus_oligofermentans
Streptococcus_gordonii
Streptococcus_sp_oral_taxon_056
Streptococcus_sanguinis
Streptococcus_mitis
Streptococcus_pneumoniae
Streptococcus_oralis
Streptococcus_sp_C300
Streptococcus_parasanguinis
Lactobacillus_crispatus
Lactobacillus_jensenii
Gemella_haemolysans
Thermobacillus_composti
Veillonella_sp_3_1_44
Veillonella_sp_6_1_27
Veillonella_parvula
Veillonella_dispar
Veillonella_atypica
Selenomonas_noxia
Selenomonas_sp_oral_taxon_892
Lachnoanaerobaculum_saburreum
Lachnospiraceae_oral_taxon_107
Lachnoanaerobaculum_sp_OBRC55
Oribacterium_sinus
Stomatobaculum_longum
Ruminococcus_sp_5_1_39BFAA
Eubacterium_rectale
Roseburia_hominis
Lachnospiraceae_bacterium_1_1_57FAA
Clostridium_sp_L250
Ruminococcus_bicirculans
Subdoligranulum_sp_4_3_54A2FAA
Eubacterium_siraeum
Actinomyces_viscosus
Actinomyces_sp_oral_taxon_175
Actinomyces_gerencseriae
Actinomyces_graevenitzii
Actinomyces_sp_oral_taxon_181
Actinomyces_sp_S6Spd3
Rothia_mucilaginosa_ATCC_25296
Rothia_mucilaginosa
Rothia_dentocariosa
Propionibacterium_acnes
Propionibacterium_propionicum
Corynebacterium_matruchotii
Atopobium_sp_ICM42b
Eubacterium_infirmum
Eubacterium_sulci_ATCC_35585
Mogibacterium_sp_CM50
Leptotrichia_sp_oral_taxon_215
Leptotrichia_buccalis
Leptotrichia_goodfellowii
Fusobacterium_nucleatum
Fusobacterium_periodonticum
Kingella_denitrificans
Neisseria_sp_oral_taxon_014
Neisseria_sicca
Neisseria_meningitidis
Neisseria_subflava
Kingella_oralis
Lautropia_mirabilis
Comamonadaceae_bacterium_H1
Haemophilus_haemolyticus
Aggregatibacter_sp_oral_taxon_458
Pasteurella_bettyae
Haemophilus_parainfluenzae
Pseudomonas_sp_BAY1663
Acinetobacter_sp_CIP_102143
Thioalkalivibrio_sulfidiphilus
Campylobacter_concisus
Bacteroides_finegoldii_CL09T03C10
Bacteroides_thetaiotaomicron
Bacteroides_salyersiae
Bacteroides_pyogenes
Bacteroides_sp_2_1_16
Bacteroides_fragilis
Bacteroides_stercorirosoris
Bacteroides_cellulosilyticus
Prevotella_sp_oral_taxon_306
Prevotella_sp_C561
Prevotella_denticola
Prevotella_oulorum
Prevotella_sp_HJM029
Prevotella_nigrescens
Prevotella_nigrescens_ATCC_33563
Prevotella_intermedia
Bacteroides_vulgatus
Bacteroides_sp_3_1_40A
Bacteroides_sp_3_1_33FAA
Parabacteroides_merdae
Parabacteroides_distasonis
Porphyromonas_sp_KLE_1280
Capnocytophaga_granulosa
Capnocytophaga_gingivalis
Capnocytophaga_sp_oral_taxon_324
0.2

Figure S2

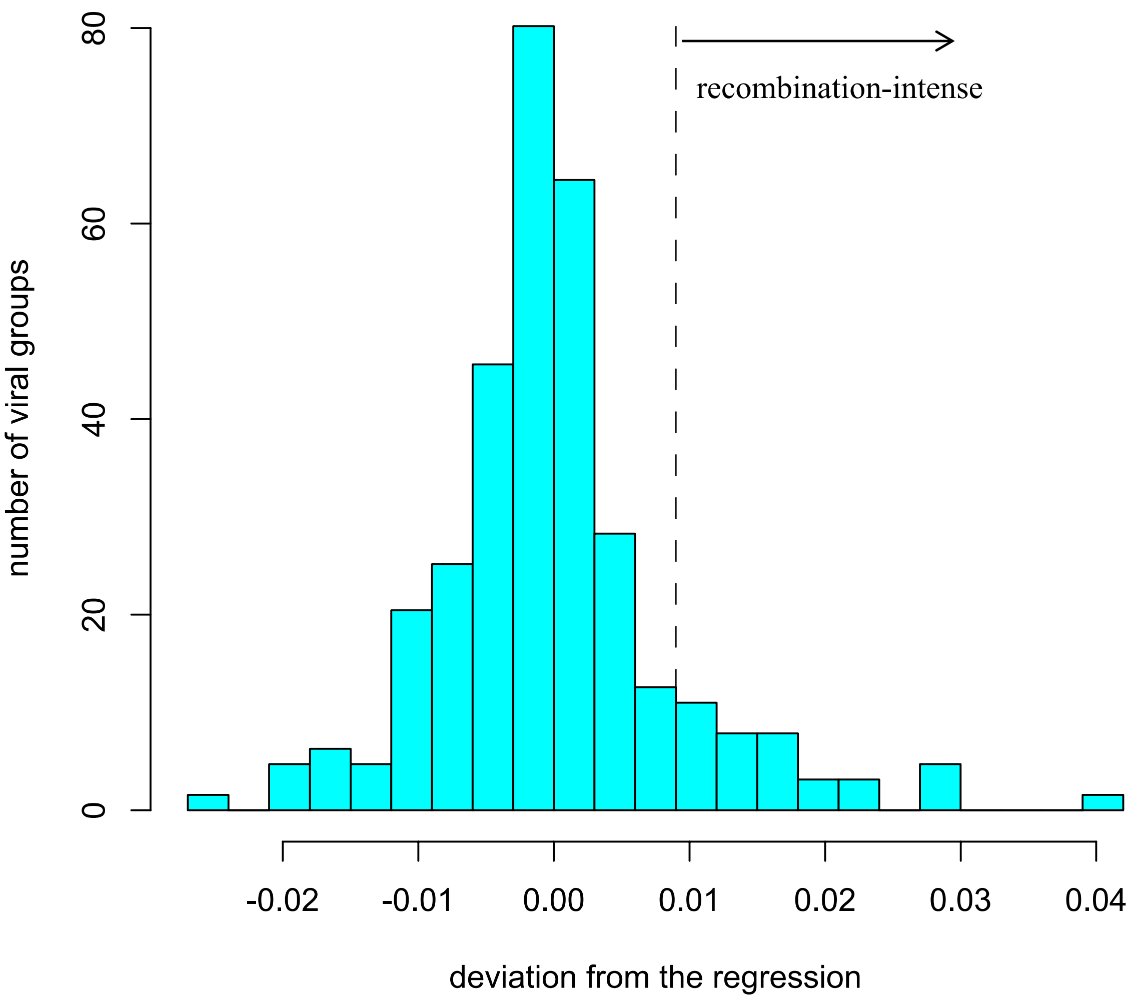
recombination-intense
number of viral groups
0
20
40
60
80
-0.02
-0.01
0.00
0.01
0.02
0.03
0.04
deviation from the regression

viralgroup 2961 (mean coverage 64.1, SD 13.8)

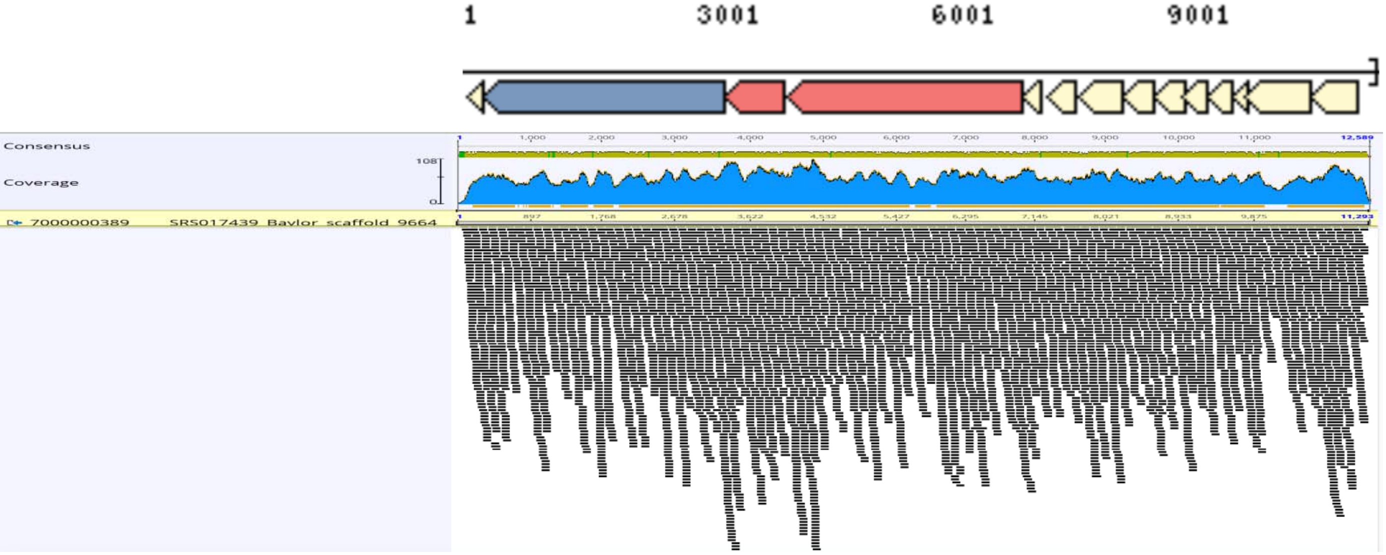

viralgroup 2959 (mean coverage 3.4, SD 2.4)

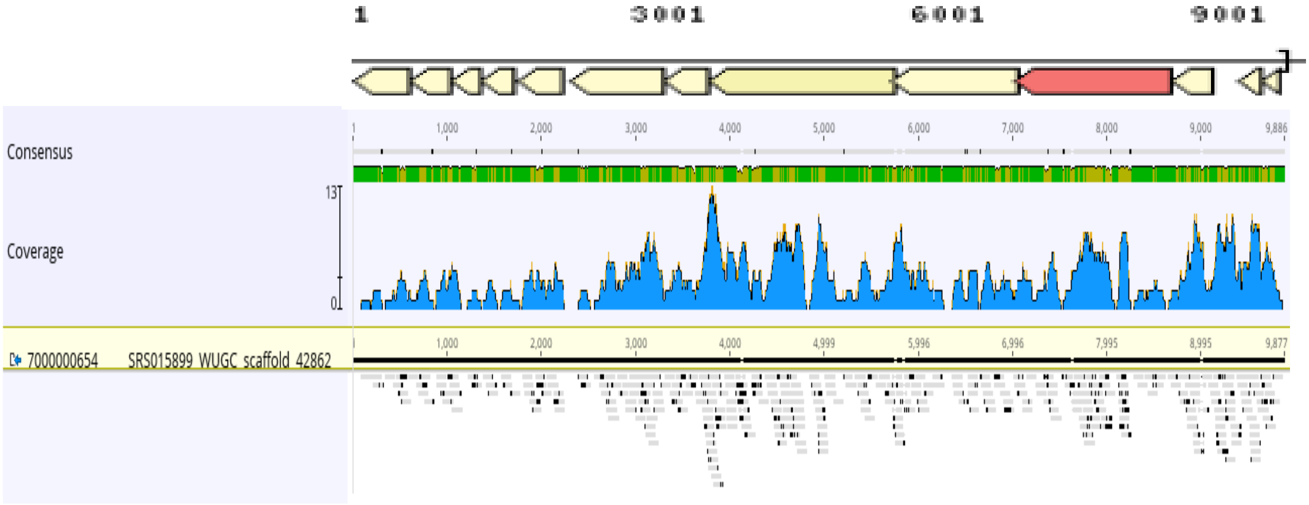

viralgroup 4460 (mean coverage 7.5, SD 4.2)

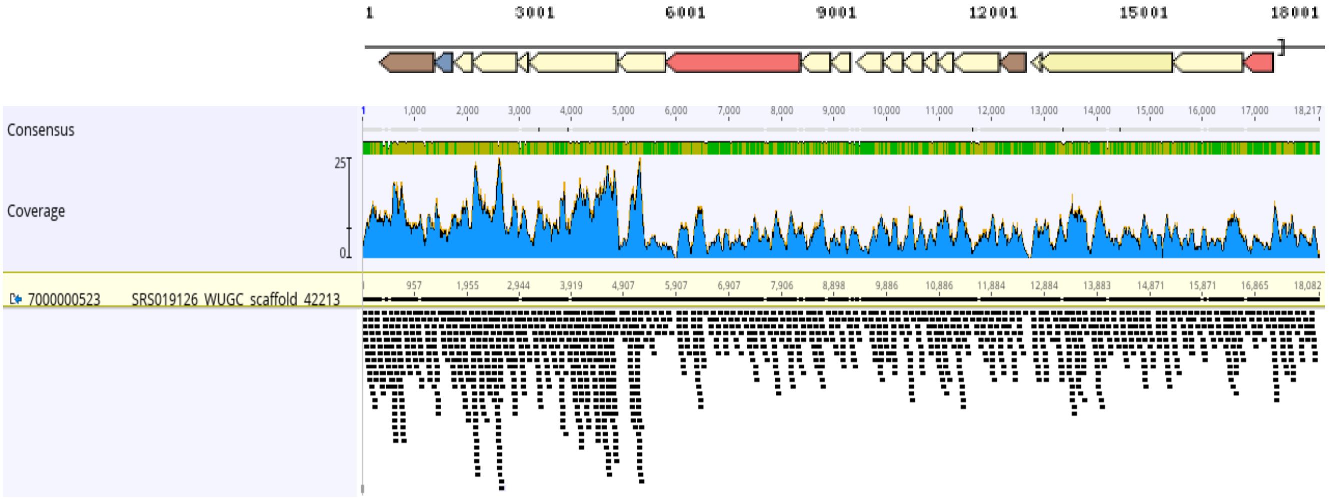

viralgroup 2890 (mean coverage 14.2, SD 5.0)

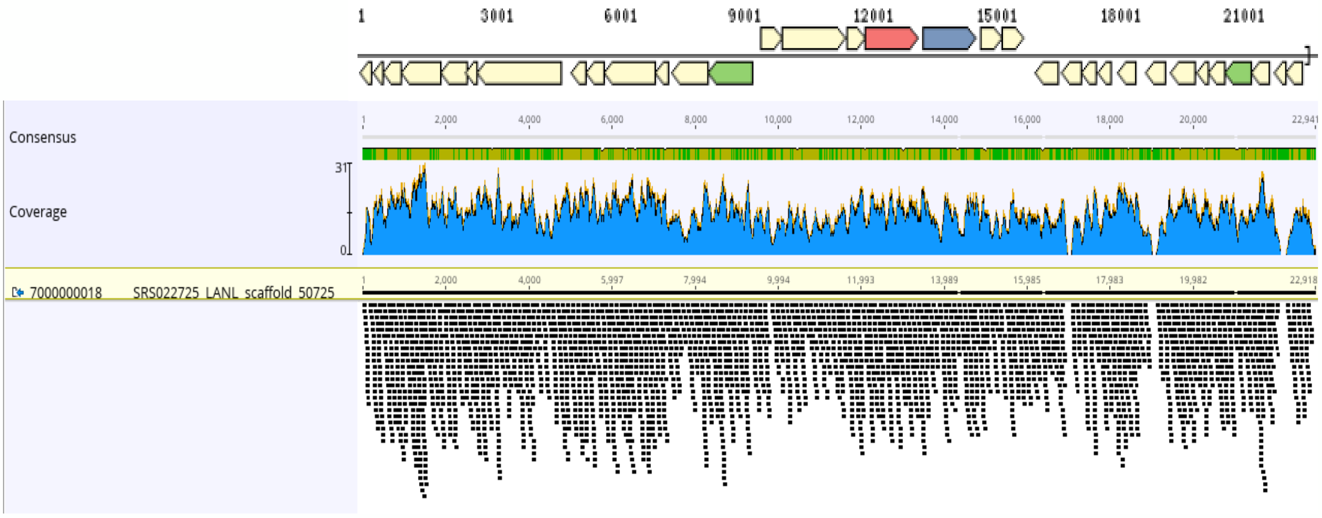

viralgroup 3707 (mean coverage 13.8, SD 6.5)

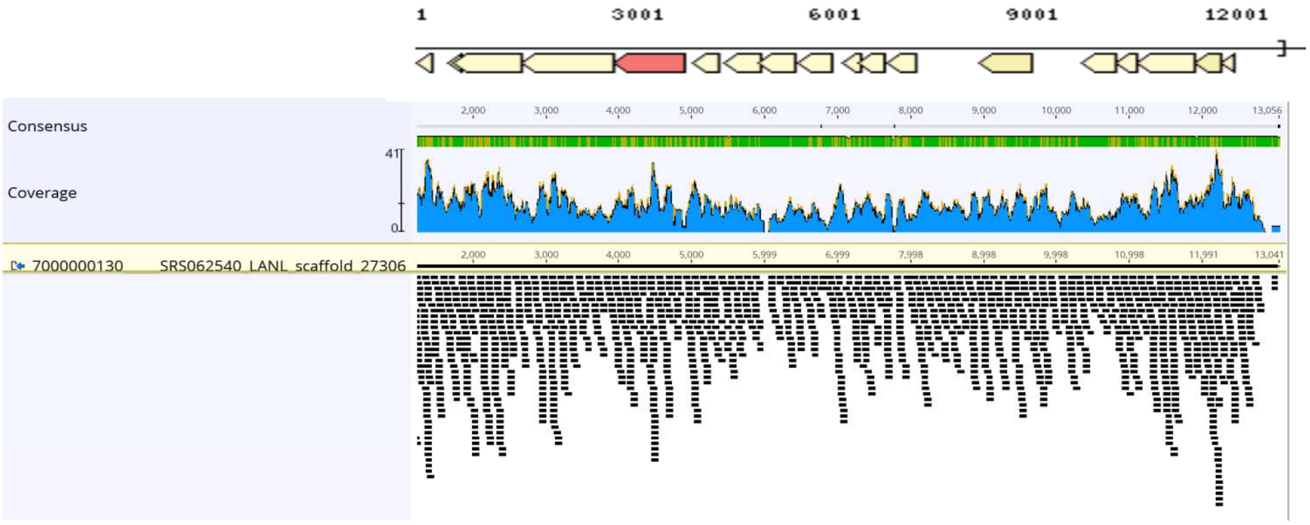

viralgroup 3184 (mean coverage 27.6, SD 14.2)

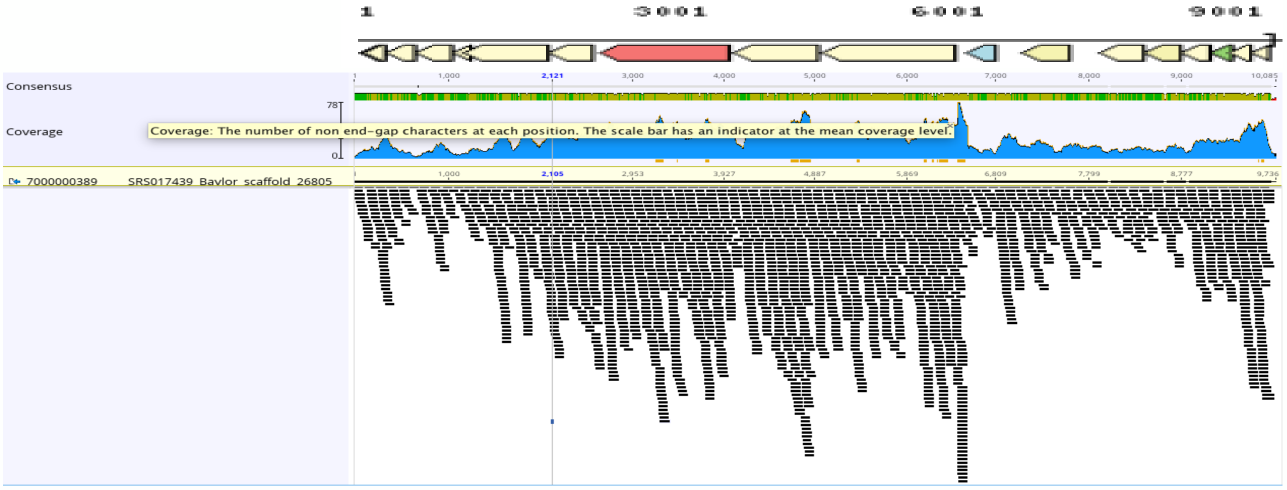

viralgroup 3319 (mean coverage 10.5, SD 4.0)

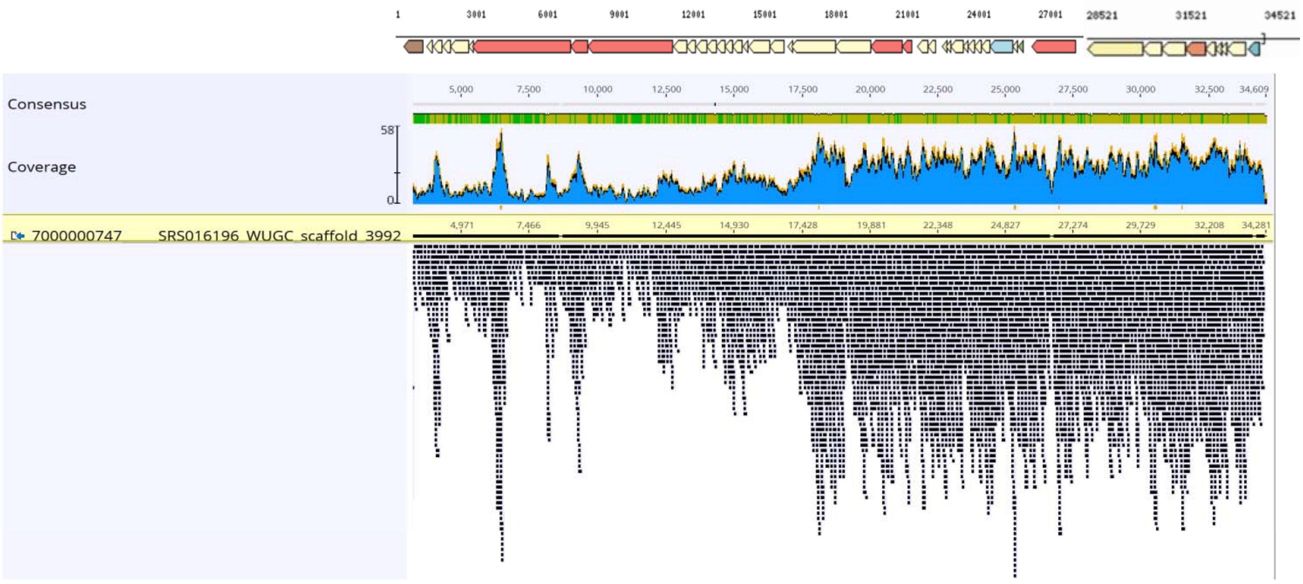

viralgroup 3776 (mean coverage 35.7, SD 8.5)

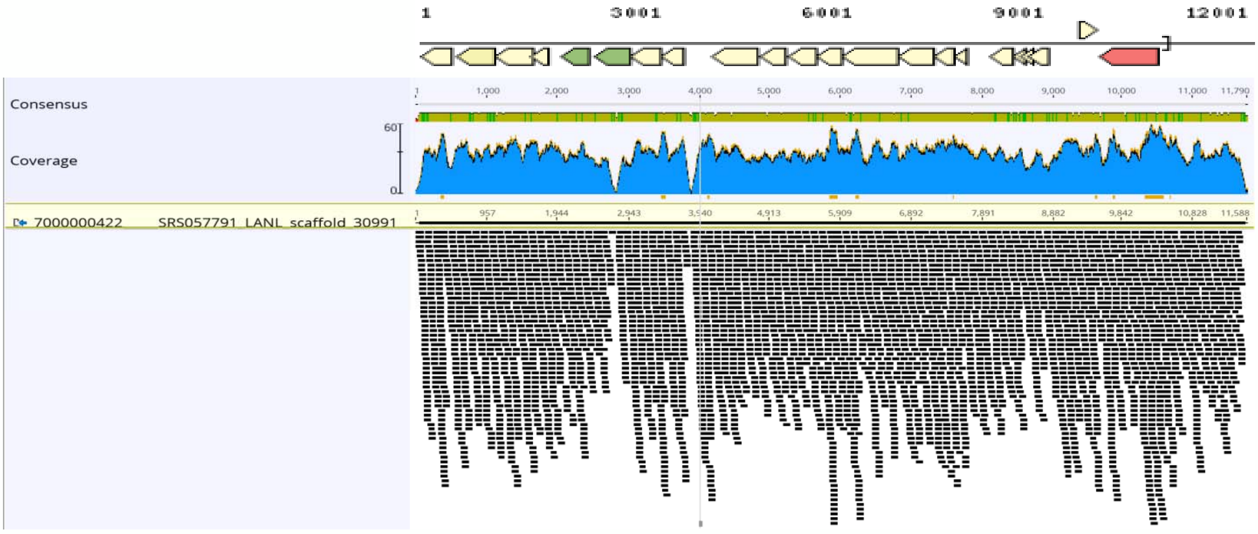

viralgroup 2970 (mean coverage 9.1, SD 3.3)

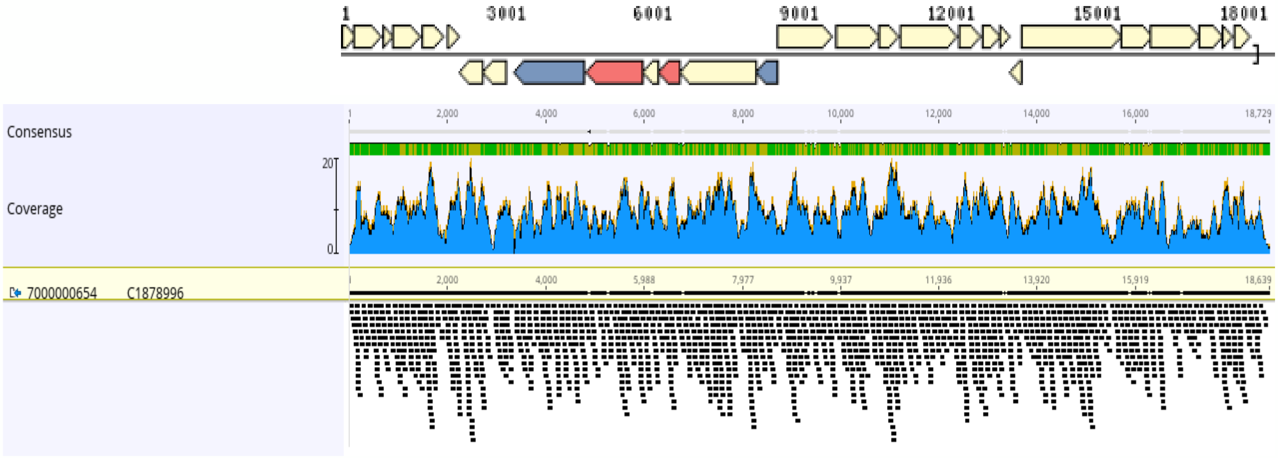

Figure S3

viralgroup 3576 (mean coverage 9.7, SD 4.3)

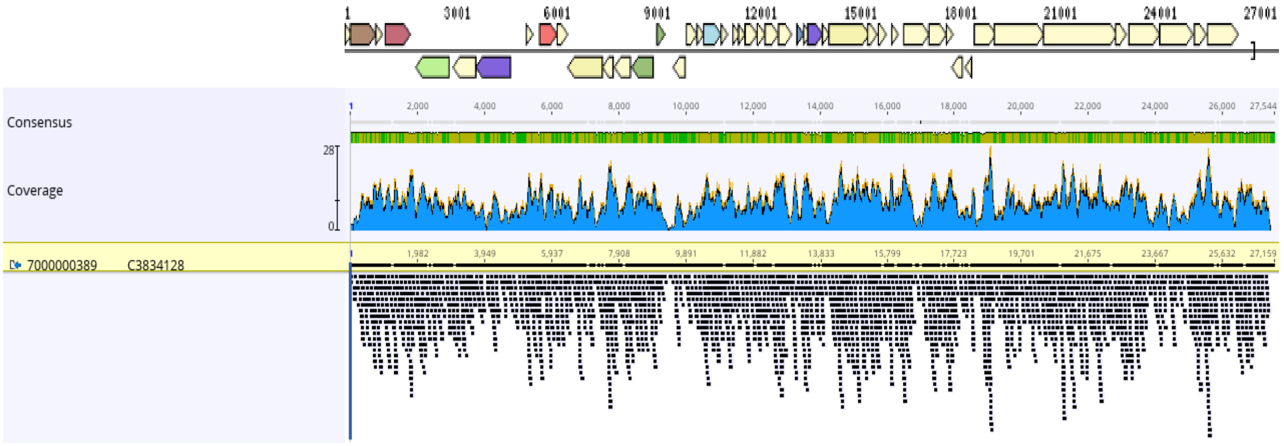

viralgroup 181 (mean coverage106.9, SD 27.8)

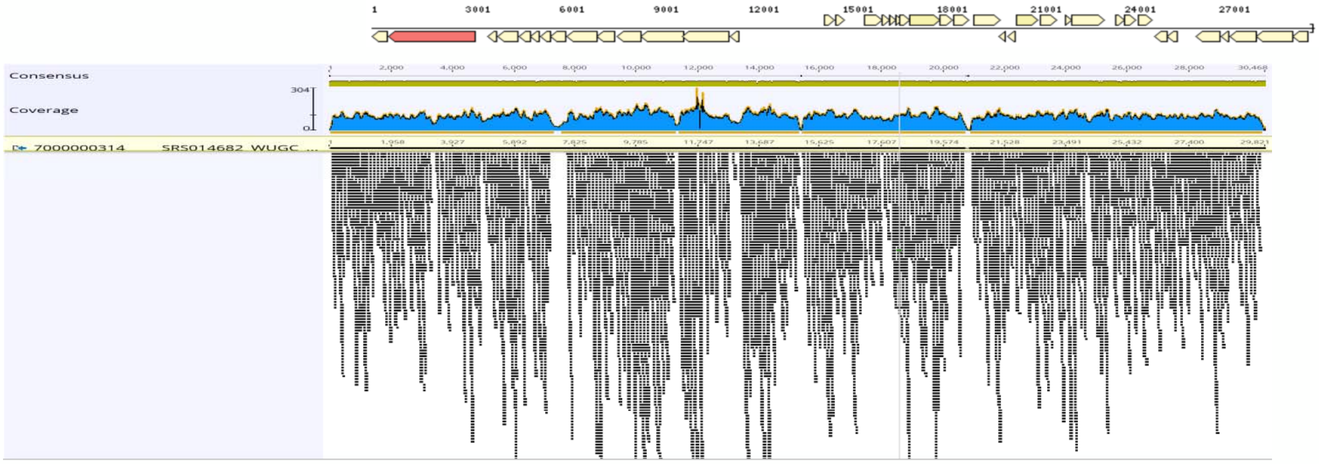

viralgroup 3189 (mean coverage 10.5, SD 4.0)

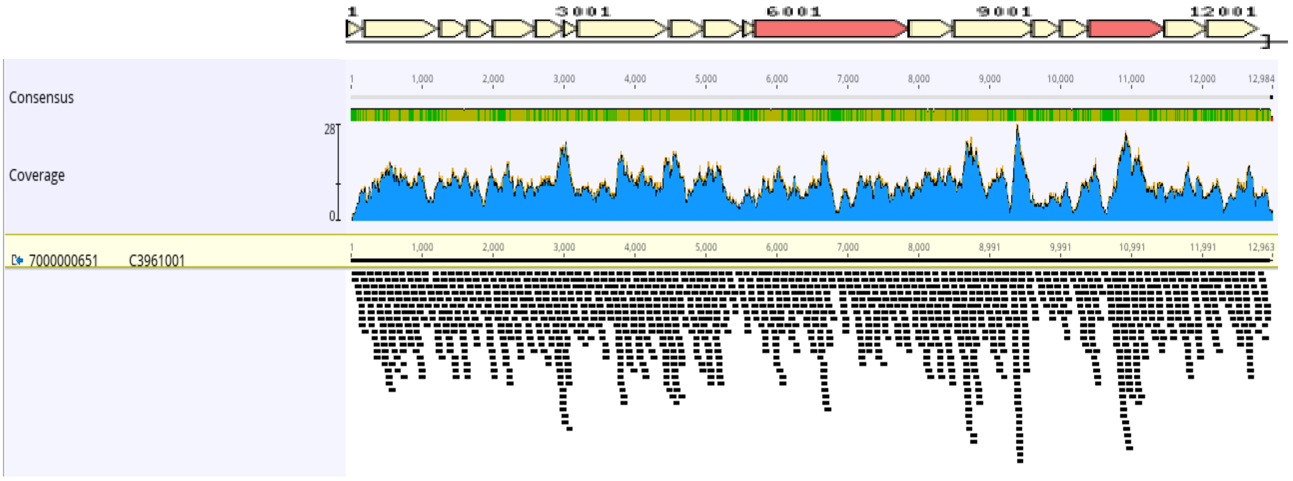

Figure S3

viralgroup 3549 (mean coverage 21.2, SD 8.2)

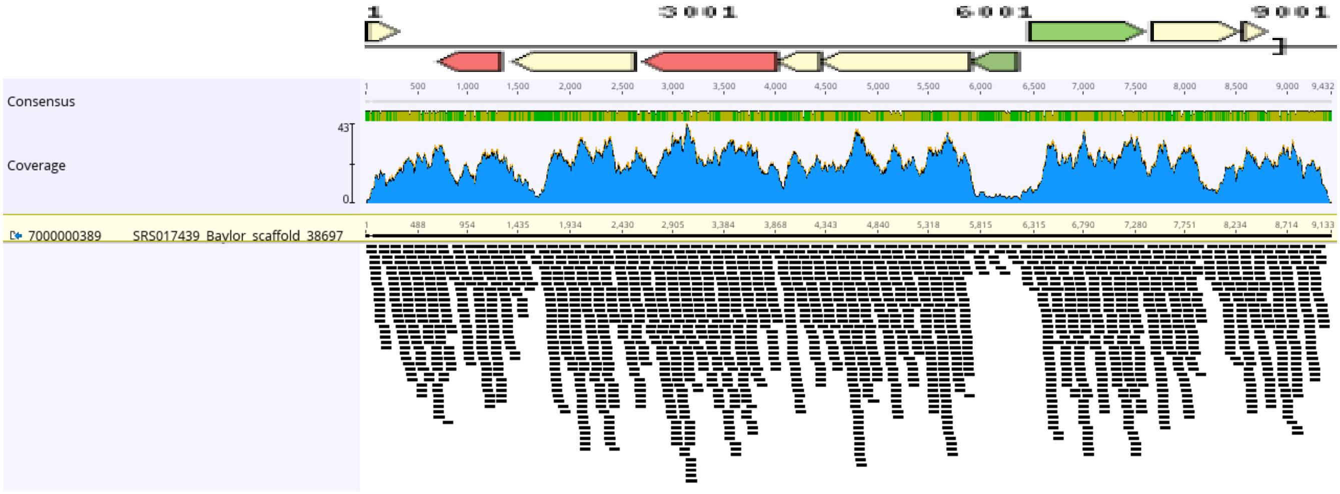

viralgroup 3600 (mean coverage 24.6, SD 9.0)

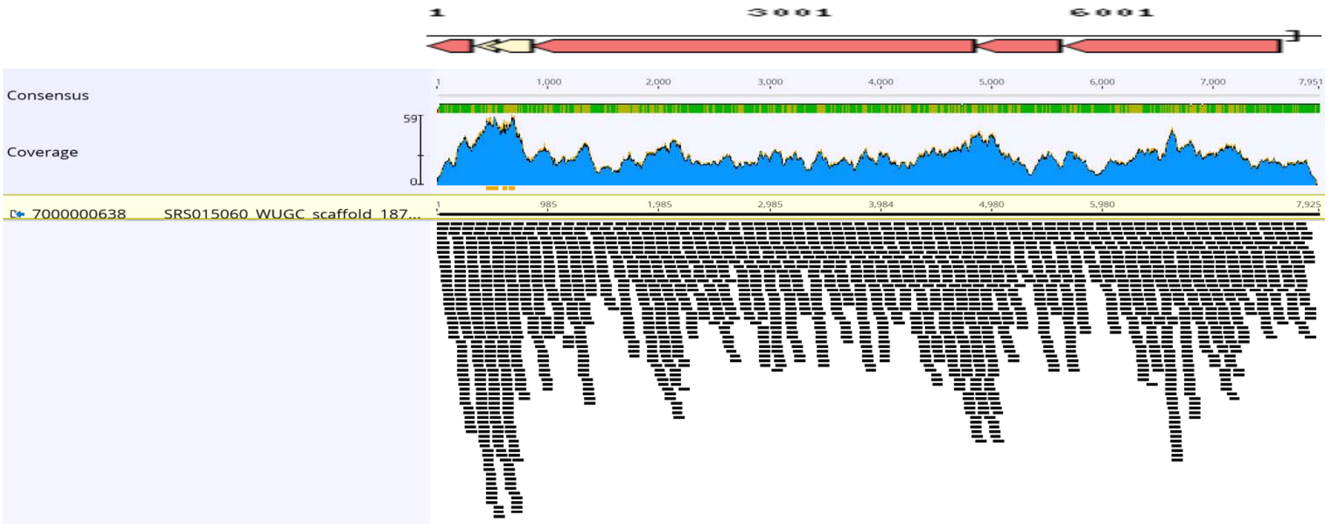

viralgroup 4000 (mean coverage 27.1, SD 12.5)

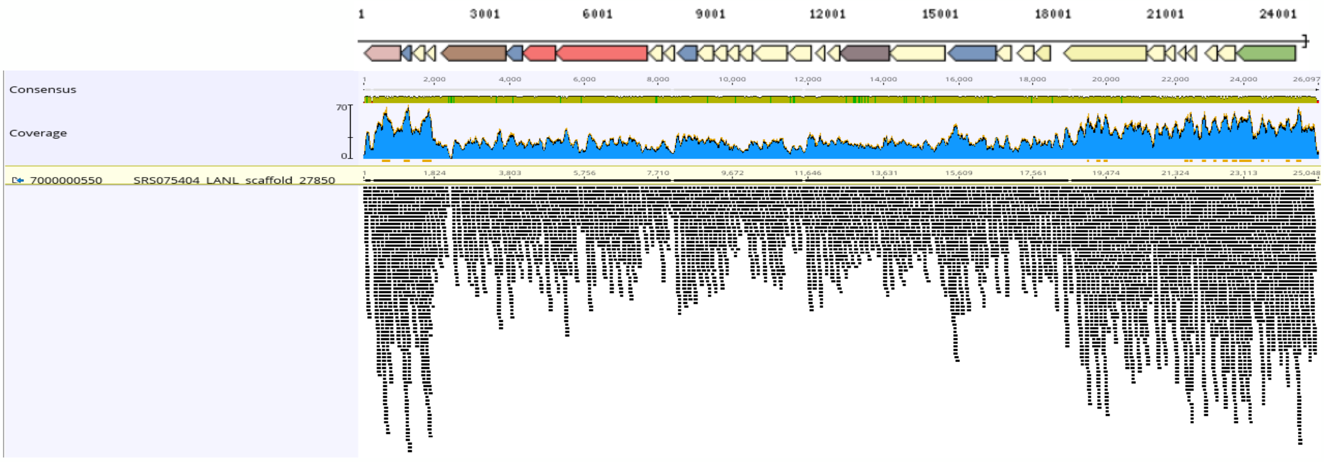

Figure S3

viralgroup 3591 (mean coverage 23.5, SD 7.3)

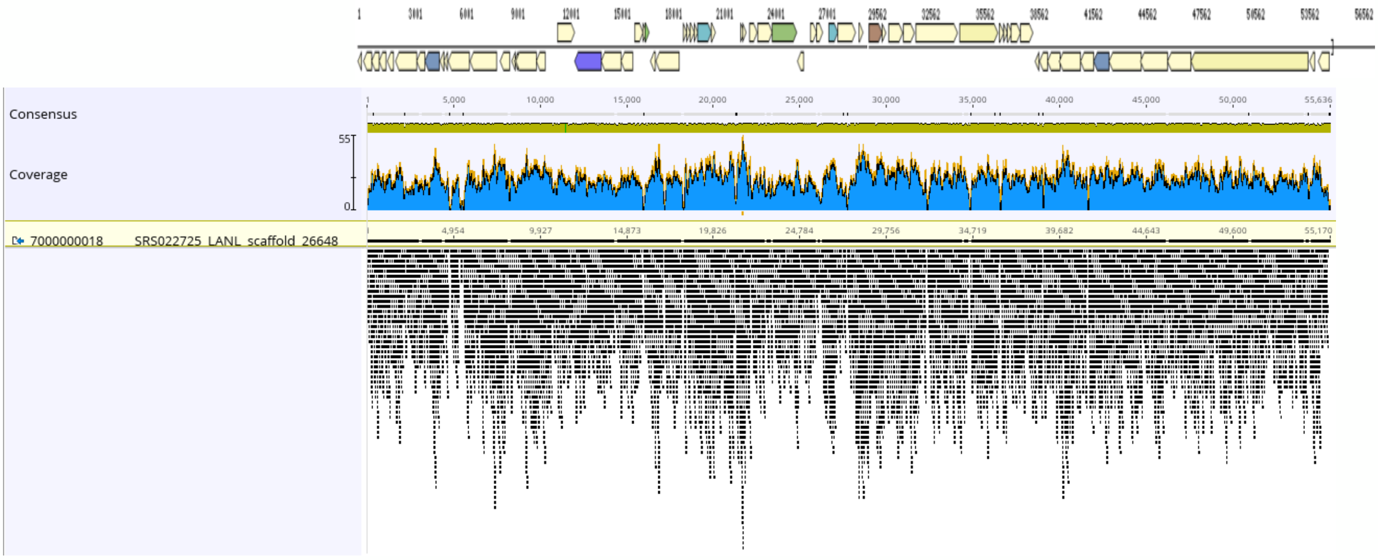

viralgroup 2981 (mean coverage 13.1, SD 4.7)

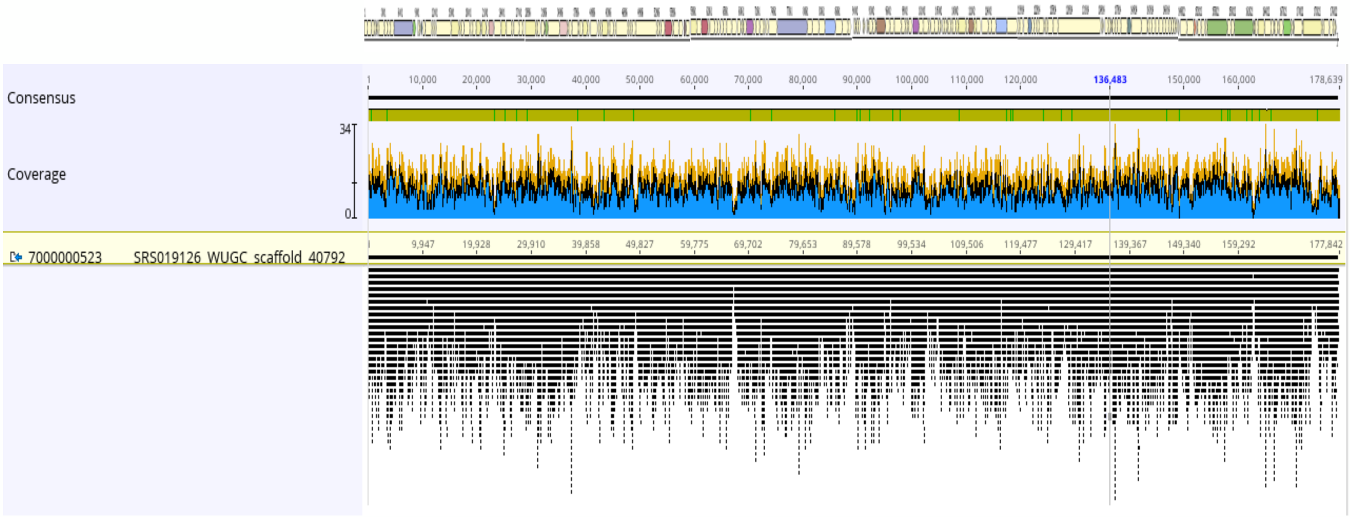

viralgroup 3836 (mean coverage 16.0, SD 4.7)

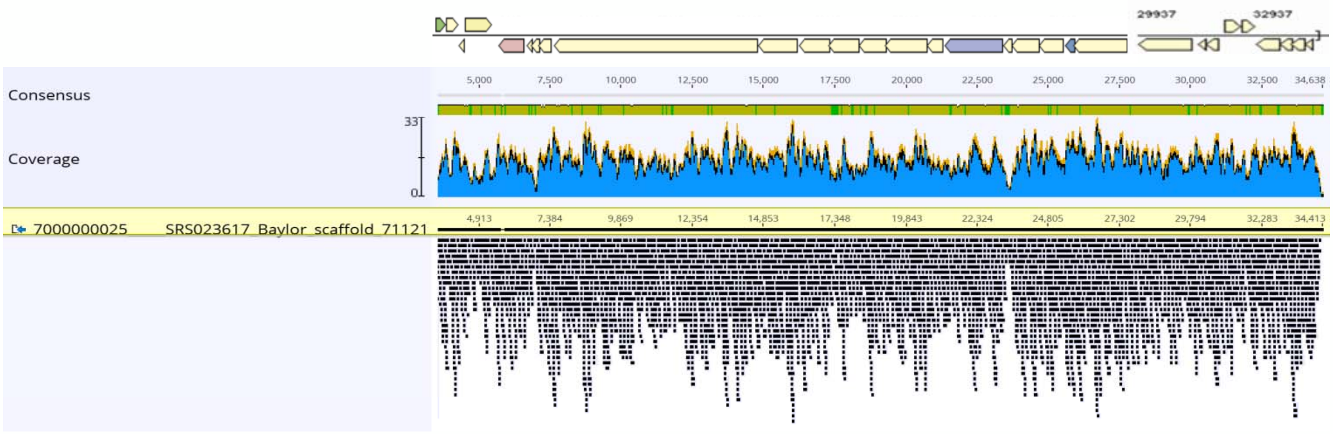

Figure S3

viralgroup 3050 (mean coverage 9.0, SD 4.2)

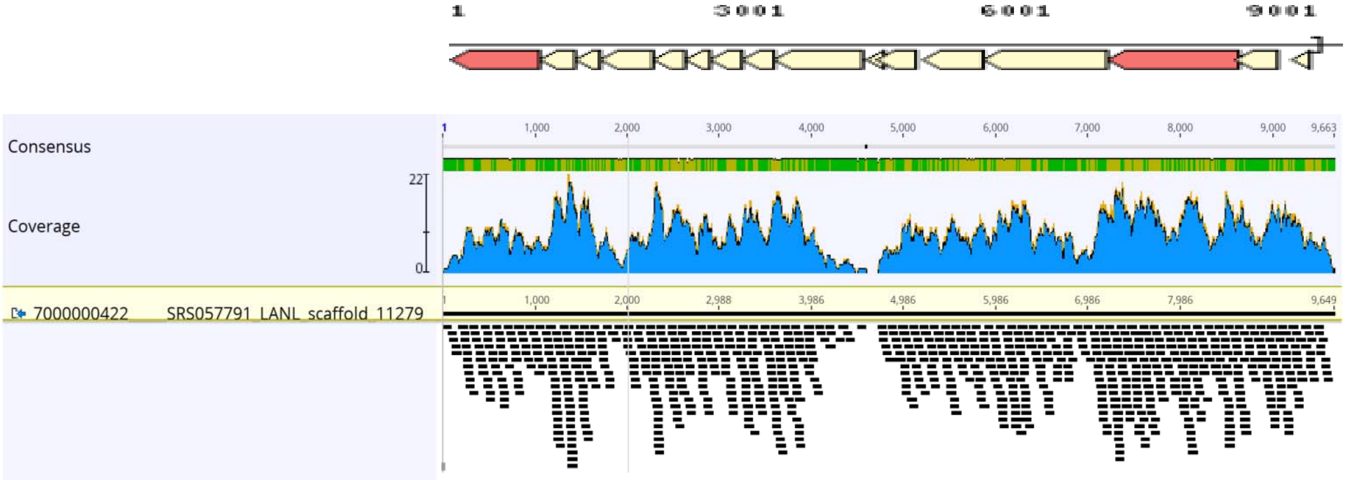

viralgroup 3445 (mean coverage 20.8, SD 5.7)

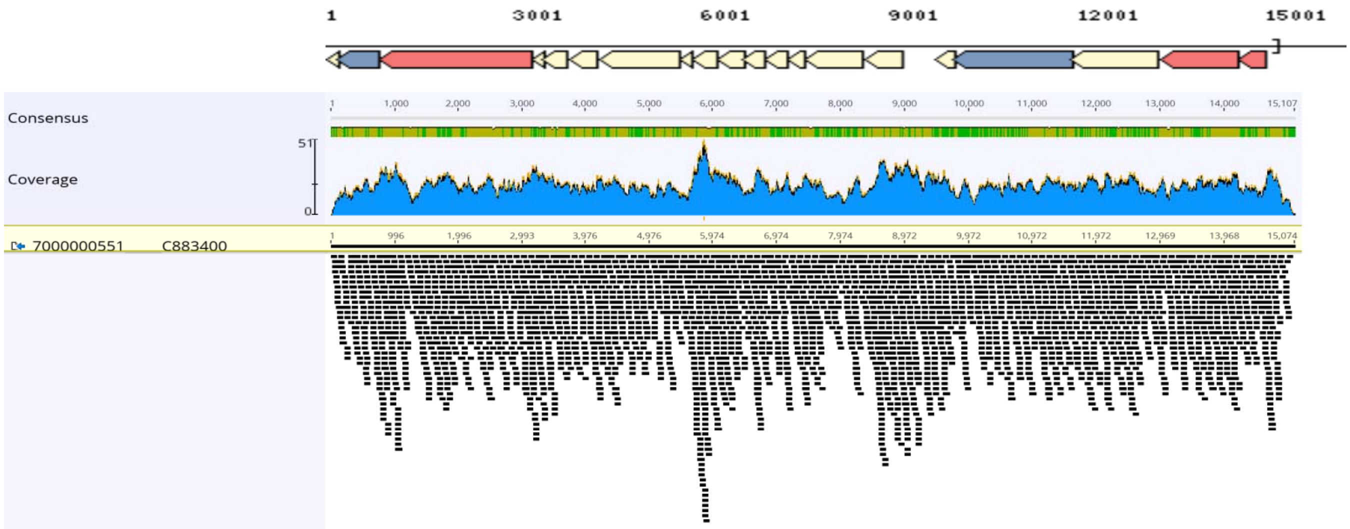

viralgroup 4484 (mean coverage 21.2, SD 8.2)

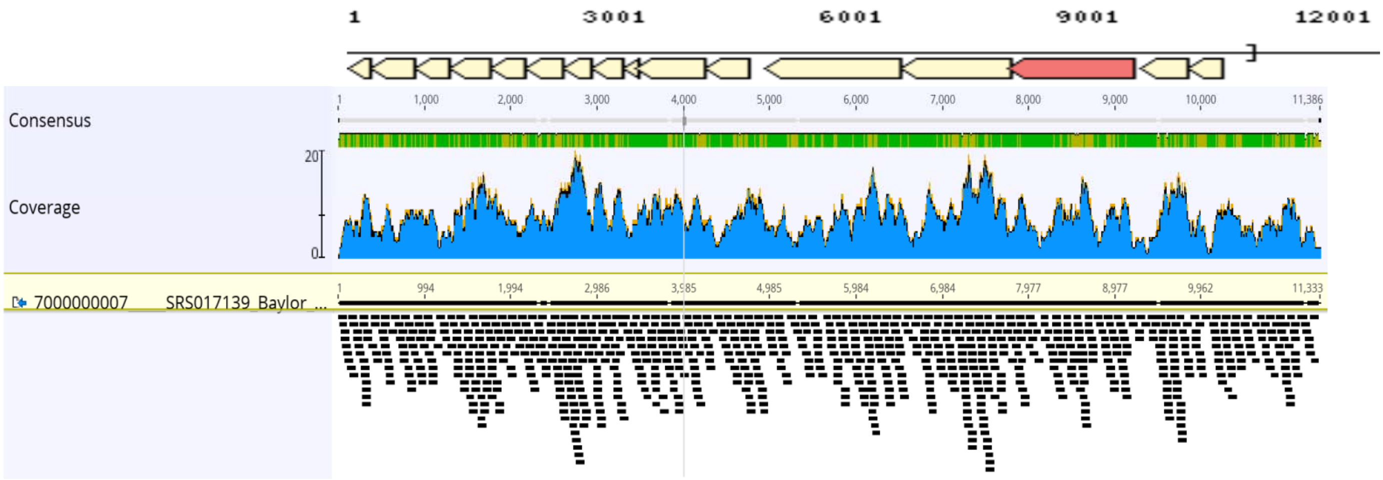

Figure S3

viralgroup 3358 (mean coverage 32.0, SD 10.9)

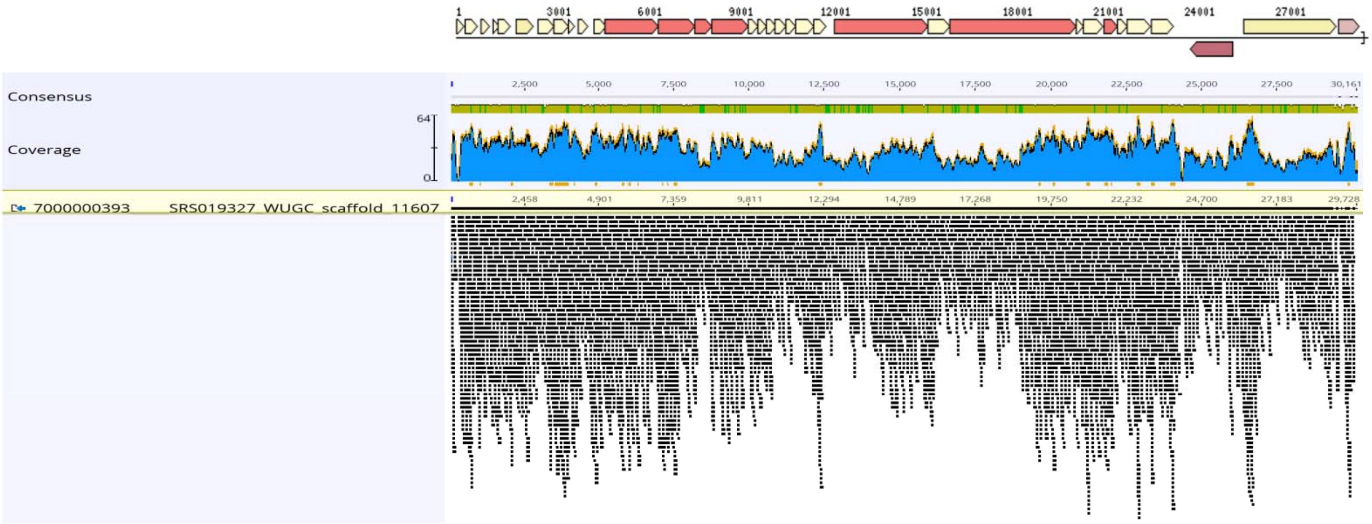

viralgroup 3915 (mean coverage 8.5, SD 4.3)

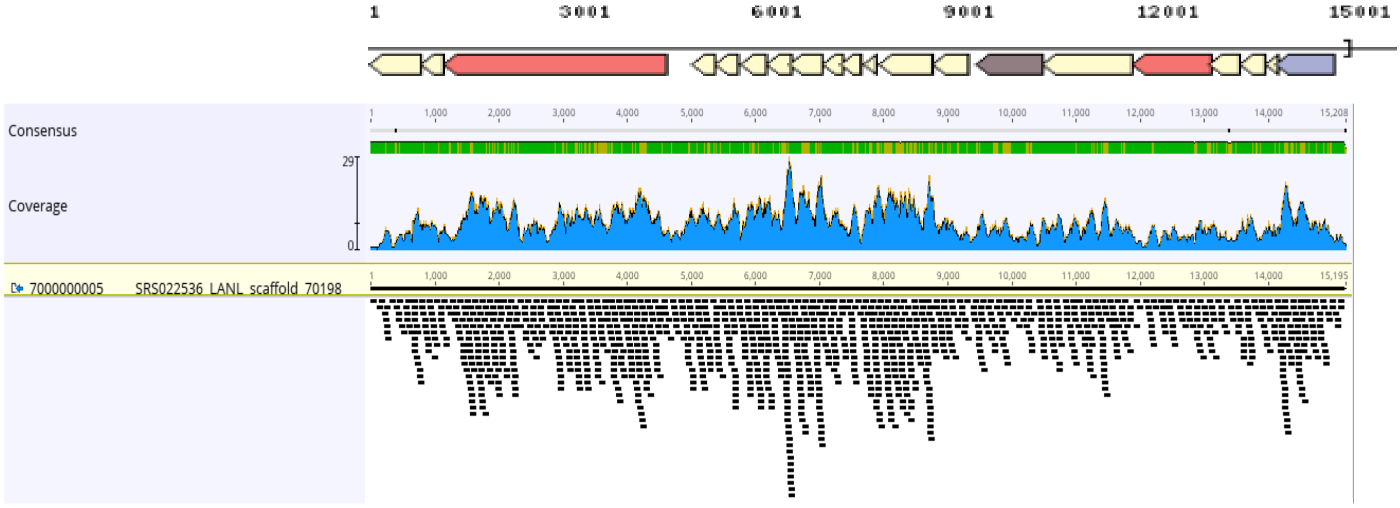

viralgroup 3200 (mean coverage 44.7, SD 33.7)

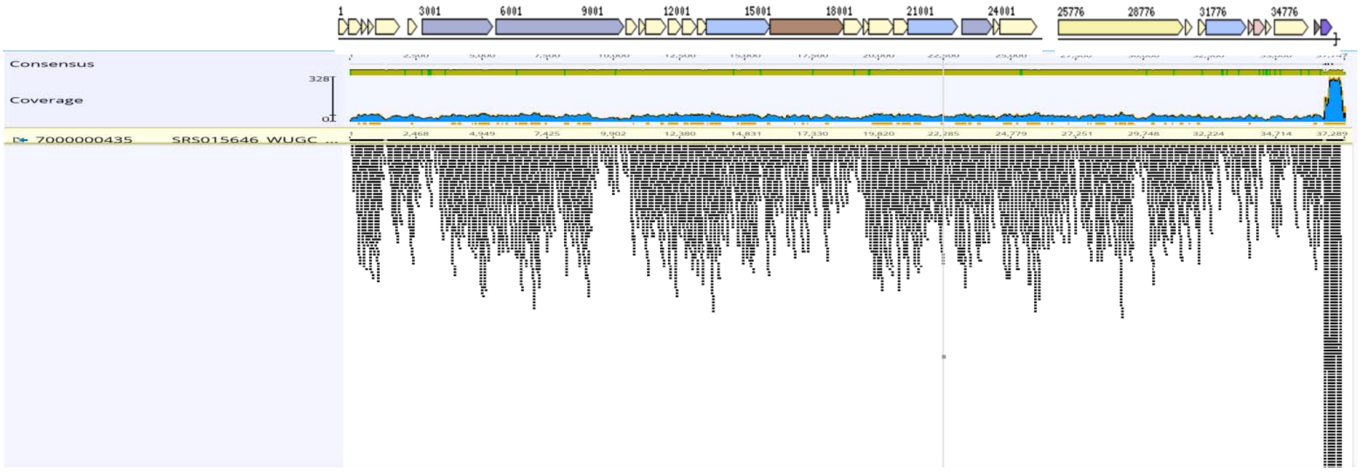

COG Coloring Selection

Color code of function category for top COG hit is shown below.
You may select a subset to view specific categories.

Show Color	Color	Description
✔	[A]	RNA processing and modification
✔	[B]	Chromatin structure and dynamics
✔	[C]	Energy production and conversion
✔	[D]	Cell cycle control, cell division, chromosome partitioning
✔	[E]	Amino acid transport and metabolism
✔	[F]	Nucleotide transport and metabolism
✔	[G]	Carbohydrate transport and metabolism
✔	[H]	Coenzyme transport and metabolism
✔	[I]	Lipid transport and metabolism
✔	[J]	Translation, ribosomal structure and biogenesis
✔	[K]	Transcription
✔	[L]	Replication, recombination and repair
✔	[M]	Cell wall/membrane/envelope biogenesis
✔	[N]	Cell motility
✔	[O]	Posttranslational modification, protein turnover, chaperones
✔	[P]	Inorganic ion transport and metabolism
✔	[Q]	Secondary metabolites biosynthesis, transport and catabolism
✔	[R]	General function prediction only
✔	[S]	Function unknown
✔	[T]	Signal transduction mechanisms
✔	[U]	Intracellular trafficking, secretion, and vesicular transport
✔	[V]	Defense mechanisms
✔	[W]	Extracellular structures
✔	[X]	Mobilome: prophages, transposons
✔	[Y]	Nuclear structure
✔	[Z]	Cytoskeleton

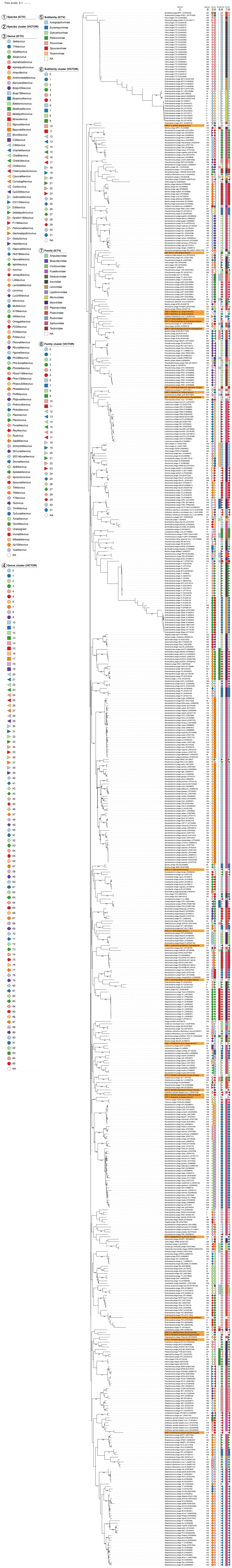
Tree scale: 0.1
1 Species (ICTV)
2 Species cluster (VICTOR)
3 Genus (ICTV)
3alikevirus
77likevirus
Ahjdlikevirus
Allolevivirus
Alphafusellovirus
Alphalipothrixvirus
Ampullavirus
Andromedalikevirus
Barnyardlikevirus
Bcep22likevirus
Bcep78likevirus
Bcepmulikevirus
Bdellomicrovirus
Betafusellovirus
Betalipothrixvirus
Bicaudavirus
Bignuzlikevirus
Bppunalikevirus
Bronlikevirus
C2likevirus
C5likevirus
Charlielikevirus
Che8likevirus
Che9clikevirus
Chilikevirus
Chlamydiamicrovirus
Cjwunalikevirus
Corndoglikevirus
Corticovirus
Cp220likevirus
Cp8unalikevirus
D3112likevirus
D3likevirus
Deltalipothrixvirus
Epsilon15likevirus
F116likevirus
Felixounalikevirus
Gammalipothrixvirus
Globulovirus
Halolikevirus
Hapunalikevirus
Hk578likevirus
Hpunalikevirus
Iebhlikevirus
Inovirus
Jerseylikevirus
L5likevirus
Lambdalikevirus
Levivirus
Luz24likevirus
Microvirus
Mulikevirus
N15likevirus
N4likevirus
Omegalikevirus
P22likevirus
P23likevirus
P2likevirus
Pbiunalikevirus
Pbunalikevirus
Pgonelikevirus
Phi29likevirus
Phic3unalikevirus
Phicbklikevirus
Phicd119likevirus
Phie125likevirus
Phieco32likevirus
Phietalikevirus
Phifllikevirus
Phijlunalikevirus
Phikmvlikevirus
Phikzlikevirus
Plasmavirus
Plectrovirus
Punalikevirus
Reylikevirus
Rudivirus
Sap6likevirus
Schizot4likevirus
Sfi1unalikevirus
Sfi21dtunalikevirus
Skunalikevirus
Sp6likevirus
Spbetalikevirus
Spiromicrovirus
Spounalikevirus
T4likevirus
T5likevirus
T7likevirus
Tectivirus
Tm4likevirus
Tp2unalikevirus
Tunalikevirus
Twortlikevirus
Unassigned
Viunalikevirus
Wbetalikevirus
Xp10likevirus
Yualikevirus
NA
4 Genus cluster (VICTOR)
0 1 2 3 4 5 6 7 8 9 10 11 12 13 14 15 16 17 18 19 20 21 22 23 24 25 26 27 28 29 30 31 32 33 34 35 36 37 38 39 40 41 42 43 44 45 46 47 48 49 50 51 52 53 54 55 56 57 58 59 60 61 62 63 64 65 66 67 68 69 70 71 72 73 74 75 76 77 78 79 80 81 82 83 84 85 86 87 88 89 90 91 92 93 94 95 NA
5 Subfamily (ICTV)
Autographivirinae
Eucampyvirinae
Gokushovirinae
Peduovirinae
Picovirinae
Spounavirinae
Tevenvirinae
NA
6 Subfamily cluster (VICTOR)
0 1 2 3 4 5 6 7 8 9 10 11 12 13 14 15 16 17 18 19 20 21 22 23 24 25 26 27 28 29 30 31 NA
7 Family (ICTV)
Ampullaviridae
Bicaudaviridae
Corticoviridae
Fuselloviridae
Globuloviridae
Inoviridae
Leviviridae
Lipothrixviridae
Microviridae
Myoviridae
Plasmaviridae
Podoviridae
Rudiviridae
Siphoviridae
Tectiviridae
NA
8 Family cluster (VICTOR)
0 1 2 3 4 5 6 7 8 9 10 11 12 13 14 15 16 17 18 19 20 21 22 23 24 25 26 27 28 29 30 31 NA

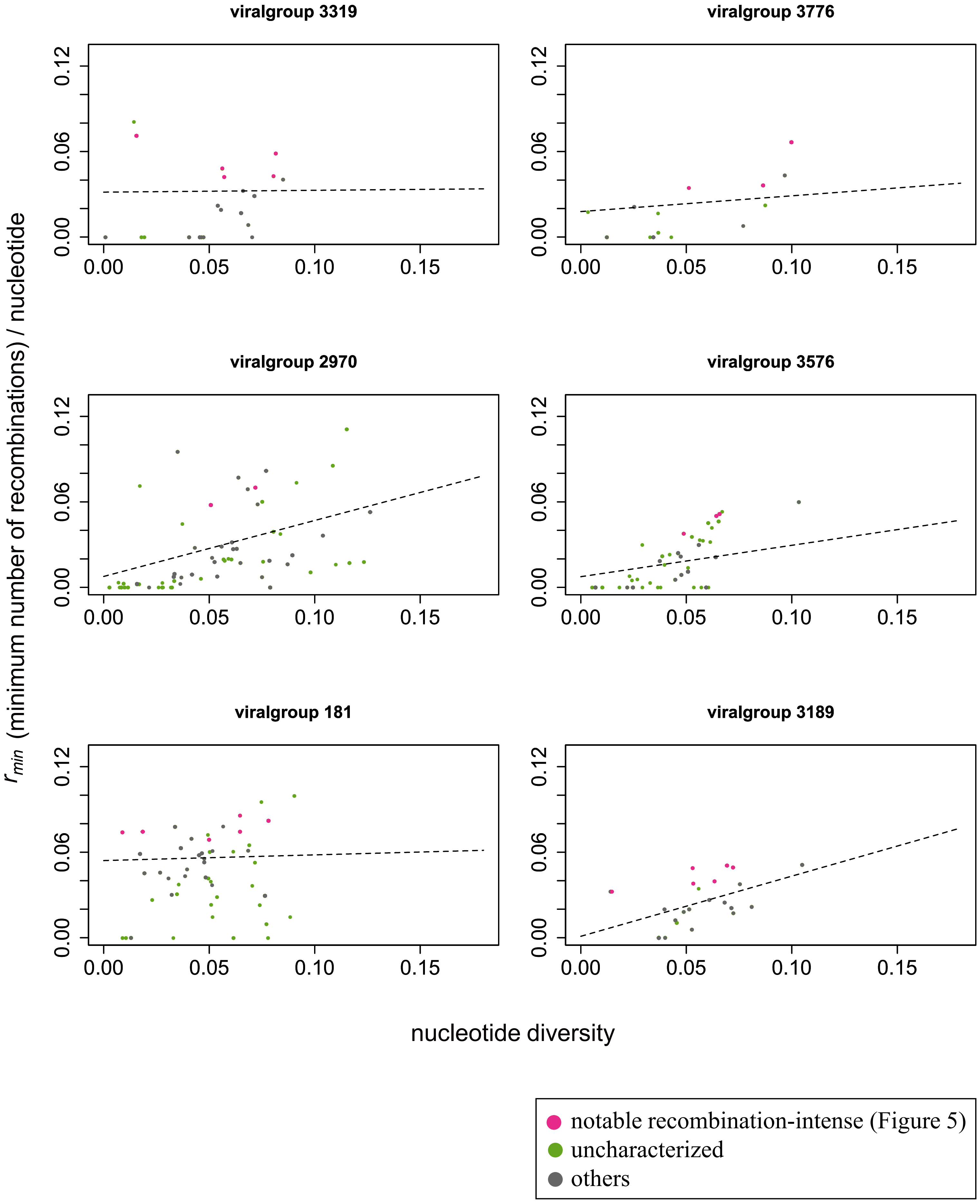
viralgroup 3319
viralgroup 3776
viralgroup 2970
viralgroup 3576
viralgroup 181
viralgroup 3189
0.00
0.06
0.12
0.00
0.05
0.10
0.15
r_{min} (minimum number of recombinations) / nucleotide
nucleotide diversity
notable recombination-intense (Figure 5)
uncharacterized
others

Figure S5

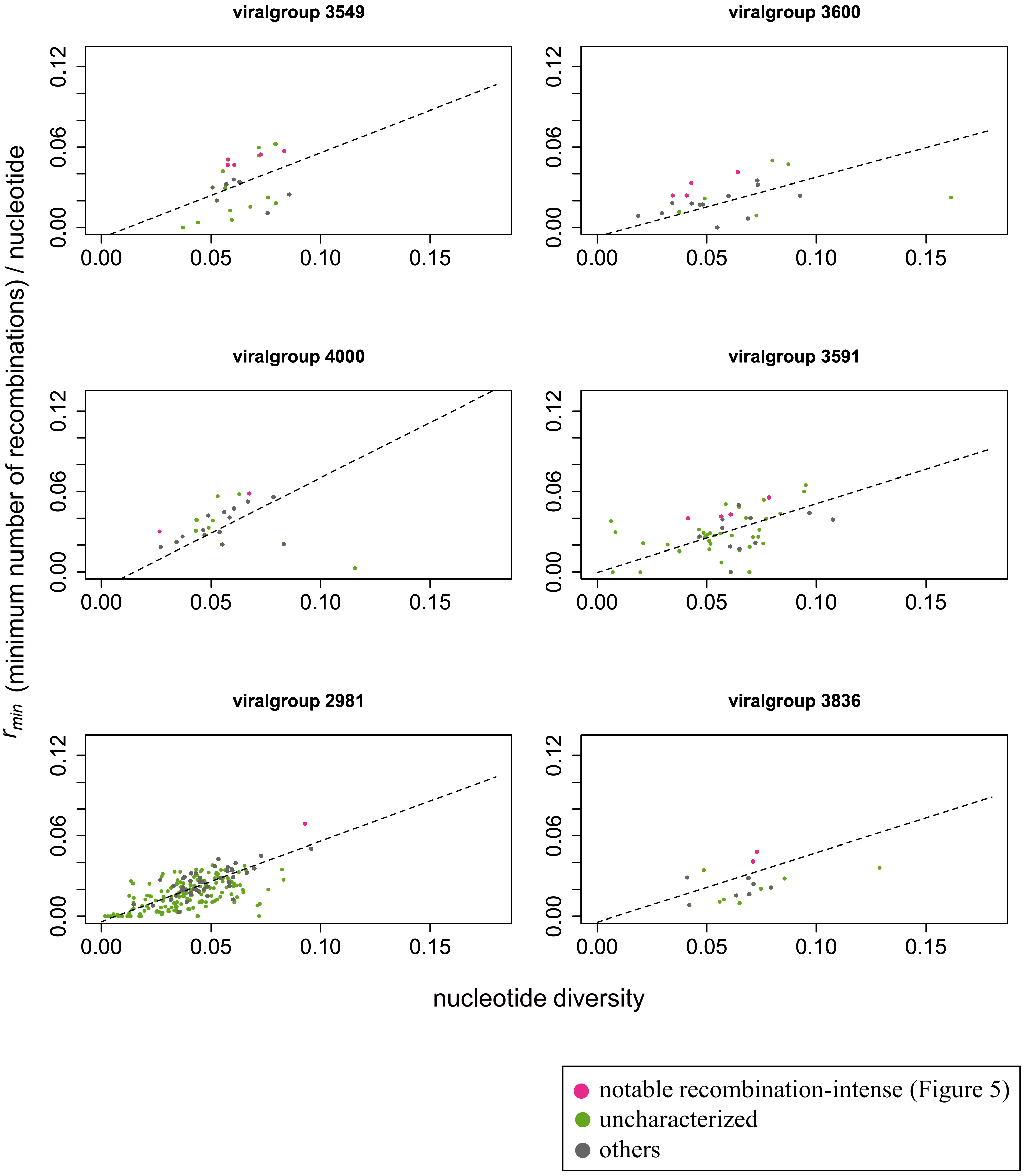
viralgroup 3549
viralgroup 3600
viralgroup 4000
viralgroup 3591
viralgroup 2981
viralgroup 3836
0.00
0.06
0.12
0.00
0.05
0.10
0.15
r_{min} (minimum number of recombinations) / nucleotide
nucleotide diversity
notable recombination-intense (Figure 5)
uncharacterized
others

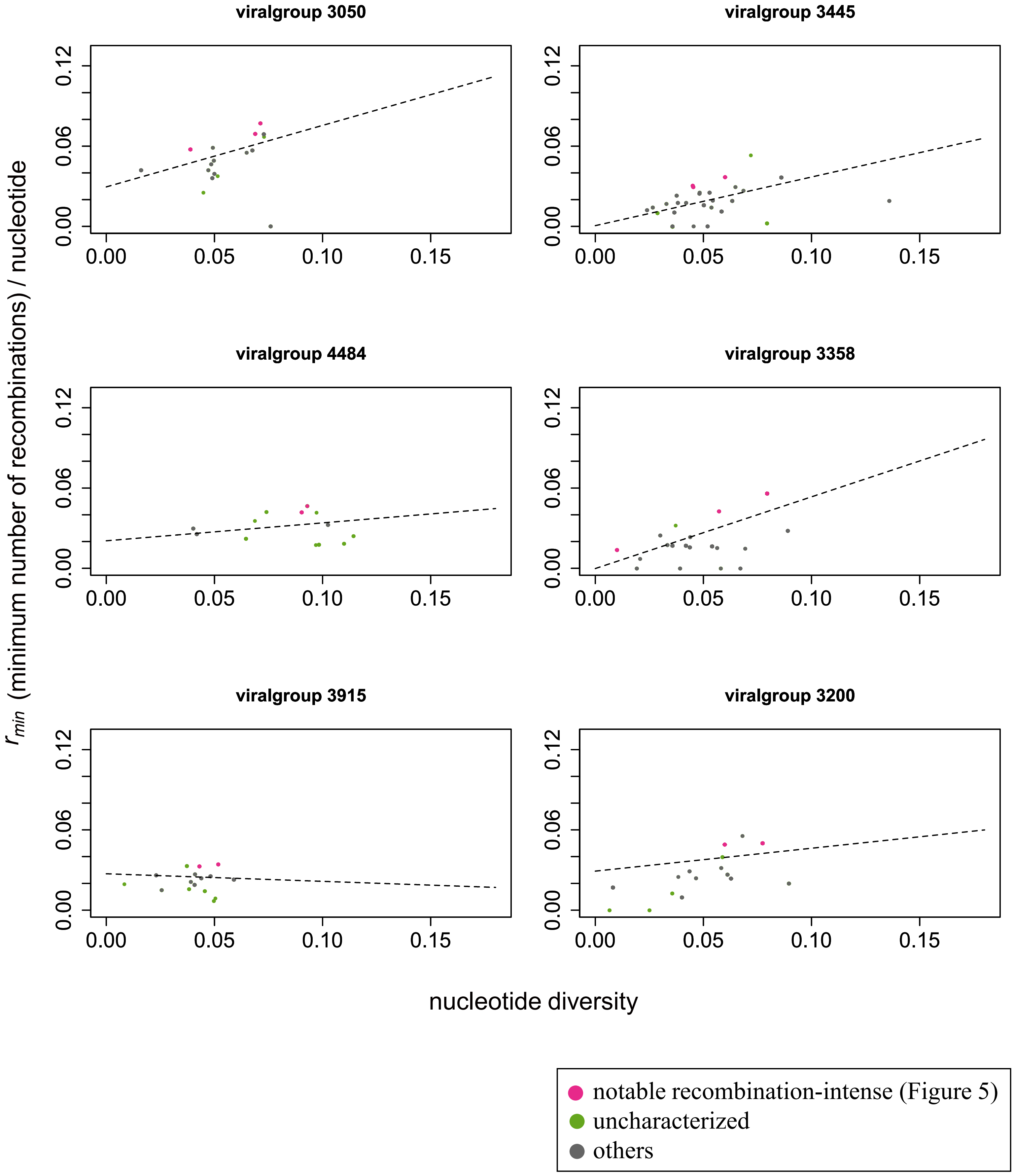
viralgroup 3050
viralgroup 3445
viralgroup 4484
viralgroup 3358
viralgroup 3915
viralgroup 3200
0.00
0.06
0.12
0.00
0.05
0.10
0.15
r_{min} (minimum number of recombinations) / nucleotide
nucleotide diversity
notable recombination-intense (Figure 5)
uncharacterized
others
